# Characterization of pathogenic genetic variants in Russian patients with primary ciliary dyskinesia using gene panel sequencing and transcript analysis

**DOI:** 10.1186/s13023-024-03318-3

**Published:** 2024-08-23

**Authors:** Anna Zlotina, Svetlana Barashkova, Sergey Zhuk, Rostislav Skitchenko, Dmitrii Usoltsev, Polina Sokolnikova, Mykyta Artomov, Svetlana Alekseenko, Tatiana Simanova, Maria Goloborodko, Olga Berleva, Anna Kostareva

**Affiliations:** 1 Almazov National Medical Research Centre, Saint-Petersburg, Russia 197341; 2 K.A. Raukhfus Children’s City Multidisciplinary Clinical Center for High Medical Technologies, Saint-Petersburg, Russia 191036; 3 Institute for Genomic Medicine, Nationwide Children’s Hospital, Columbus, OH 43205 USA; 4 Department of Pediatrics, The Ohio State University College of Medicine, Columbus, OH 43215 USA; 5 Republican Children’s Clinical Hospital of the Ministry of Health of the Udmurt Republic, Izhevsk, Russia 426009; 6 Department of Women’s and Children’s Health, Center for Molecular Medicine, Karolinska Institutet, 17176 Stockholm, Sweden

**Keywords:** Primary ciliary dyskinesia, Russian population, Gene-panel sequencing, *DNAH5*, *HYDIN*, *ZMYND10*, Splice site variants, Transcript analysis, Founder mutation

## Abstract

**Background:**

Primary ciliary dyskinesia (PCD) is a group of rare genetically heterogeneous disorders caused by defective cilia and flagella motility*.* The clinical phenotype of PCD patients commonly includes chronic oto-sino-pulmonary disease, infertility, and, in about half of cases, laterality defects due to randomization of left–right body asymmetry. To date, pathogenic variants in more than 50 genes responsible for motile cilia structure and assembly have been reported in such patients. While multiple population-specific mutations have been described in PCD cohorts from different countries, the data on genetic spectrum of PCD in Russian population are still extremely limited.

**Results:**

The present study provides a comprehensive clinical and genetic characterization of 21 Russian families with PCD living in various country regions. Anomalies of ciliary beating in patients` respiratory epithelial cells were confirmed by high-speed video microscopy. In the most cases, custom-designed panel sequencing allowed to uncover causative variants in well-known or rarely mentioned PCD-related genes, including *DNAH5*, *DNAH11*, *CFAP300*, *LRRC6*, *ZMYND10*, *CCDC103*, *HYDIN*, *ODAD4*, *DNAL1*, and *OFD1*. The variations comprised common mutations, as well as novel genetic variants, some of which probably specific for Russian patients. Additional targeted analysis of mRNA transcripts from ciliated cells enabled us to specify functional effects of newly identified genetic variants in *DNAH5* (c.2052+3G>T, c.3599-2A>G), *HYDIN* (c.10949-2A>G, c.1797C>G), and *ZMYND10* (c.510+1G>C) on splicing process. In particular, the splice site variant c.2052+3G>T, detected in four unrelated families, resulted in skipping of exon 14 in DNAH5 transcripts and, according to haplotype analysis of affected probands, was proposed as an ancestral founder mutation in Udmurt population.

**Conclusions:**

The reported data provide a vital insight into genetic background of primary ciliary dyskinesia in the Russian population. The findings clearly illustrate the utility of gene panel sequencing coupled with transcriptional analysis in identification and clinical interpretation of novel genetic variants.

**Supplementary Information:**

The online version contains supplementary material available at 10.1186/s13023-024-03318-3.

## Background

Primary ciliary dyskinesia (PCD) is a group of rare inherited disorders (~ 1/10,000–1/30,000 live birth) caused by dysfunction of motile cilia and flagella [1, 2]. While flagella ensure sperm motility, motile cilia represent tiny organelles which protrude from the cell surface in mammalian epithelium of the respiratory tract thus providing mucociliary clearance and realizing mechano-/chemosensory functions [3, 4]. Besides, multi-ciliated cells constitute the oviduct epithelium and the ependyma lining the brain ventricles. Accordingly, the clinical phenotype of PCD patients commonly includes recurrent respiratory disorders such as persistent rhinosinusitis, nasal polyps, otitis media, progressive bronchiectasis and pneumonia episodes, as well as male and female sub-/infertility, and rarely hydrocephalus [2, 5, 6]. Notably, motile monocilia also emerge from cells of the embryonic node, or left–right organizer, which is a transient structure ensuring the proper establishment of left–right patterning during early stages of embryogenesis [7]. In this connection, about a half of PCD patients demonstrate laterality defects—situs inversus totalis (Kartagener syndrome) or, in some cases, heterotaxy with complex cardiac malformations [6, 8].

The principal structural part of cilia, the axoneme, is made up of microtubule doublets (nine peripheral duplets with (“9 + 2”) or without (“9 + 0”) the central pair complex) and associated motor proteins [9]. By the moment, pathogenic variants in more than 50 genes responsible for motile cilia structure, assembly and function have been reported in PCD patients [10, 11]. Such a considerable genetic heterogeneity of PCD has been shown since high-throughput sequencing approaches, including gene-panel sequencing, exome and whole-genome sequencing, have been widely introduced into basic research and clinical practice [11, 12]. With a few exceptions, PCD is an autosomal recessive condition caused by homozygous or compound heterozygous mutations, with the majority of variants being identified in the genes encoding axonemal dynein motor proteins—components of outer (ODA) and inner (IDA) dynein arms and dynein arm preassembly factors. Many of these genes are large and have multiple protein-coding regions (as an example, *DNAH5* encoding a dynein heavy chain 5 of the ODA comprises 79 exons with one alternative first exon [13]). According to various estimates, only up to 50–75% of patients with a well-established PCD clinical phenotype have a confirmed genetic status [10, 11]. The remaining cases might be associated with additional yet uncharacterized genes or due to nontrivial genetic alterations in known PCD-related genes including variants in non-coding regions, synonymous exchanges, copy number variations or splice site and missense variants of uncertain clinical significance (VUSs). Further in-depth in vitro and/or in vivo functional analysis enables more accurate classification and clinical interpretation of the identified VUSs. In particular, transcriptional analysis performed by target Sanger sequencing or high-throughput NGS-based RNAseq is known to be a powerful tool to clarify the molecular consequences of the genetic variants that are in silico predicted to impact splicing through disruption of canonical splice sites or splicing regulatory elements, creation of ectopic splice sites or activation of cryptic splice sites, etc. [14–17]. As a result, such functional studies allow to specify alternative splicing events such as in-frame or out-of-frame indels of exonic/intronic sequences in mRNA transcripts, many of which result in disruption of an open reading frame of the translated protein.

Population-specific genetic investigations prove to be very helpful for characterization of genetic variability, determination of the PCD-related genes and pathogenic variants typical for a particular population, which gives opportunity to design effective targeted genetic tests. By now multiple population-specific pathogenic variants, both founder mutations and recurrent variants, underlying PCD conditions in different countries and nations have been reported [16, 18–26]. However, it should be noted that the publicly available data on PCD genetics in Russian population are currently extremely limited and fragmented, being mainly restricted to single case reports and short communications [27–33]. Here, we give a detailed clinical and genetic description of 21 Russian families diagnosed with primary ciliary dyskinesia. The custom gene-panel sequencing, in most cases, allowed to identify causative variants in well-known or rarely mentioned PCD-related genes, which included common mutations previously uncovered in other different populations and first described variants, some of which seemingly specific for Russia. Careful evaluation of ciliary beat pattern (CBP) and frequency (CBF), as well as target analysis of mRNA transcripts from the patients` respiratory multi-ciliated cells enabled us to confirm and specify functional effects of the newly identified genetic variations. Notably, we characterized, for the first time, the intronic variant c.2052+3G>T in *DNAH5* leading to abnormal splicing of transcripts and seemingly representing an ancestral founder mutation in the Udmurtia region. We believe that the reported data provide a vital insight into genetic background of primary ciliary dyskinesia in Russian population.

## Methods

### Patients cohort

A total of 21 families with a diagnosis of primary ciliary dyskinesia were enrolled in the study from different regions of the Russian Federation. The diagnostics was carried out based on careful clinical examination, results of high-speed video microscopy (HSVM) of the bronchial and/or nasal ciliated epithelium, immunofluorescence (IF) staining for ODA-specific DNAH5 protein and transmission electron microscopy of cilia axonemes, where available. The study was performed according to the Helsinki Declaration and study approval was obtained from the Institutional Ethical Review Board at the Almazov National Medical Research Centre in St. Petersburg. Written informed consent to participate was obtained from all of the participants, or from the parents of the children under the age of 18, included in the study.

### Brush biopsy and high-speed video microscopy of respiratory epithelium cells

Nasal-brush biopsy was carried out as previously described [34]. Briefly, respiratory epithelial cells were collected bilaterally from middle nasal concha using a cytobrush without prior local anaesthesia. Bronchial-brush biopsy was carried out via videobronchoscopy under general anesthesia, when necessary. The brush was rinsed in an Eppendorf with 37 °C normal saline after that the obtained cell suspension was spread on a microscope slide under a coverslip and visually evaluated by phase contrast microscopy using Nikon Eclipse E200 upright microscope (Nikon, Japan) equipped with a CFI Achromat DL 40X phase contrast objective and a high-speed camera Basler pu2500-14uc (Basler AG, Germany) set at 124 frames per second (fps). Video recording was carried out in two projections relative to ciliary motion (direct “top” and lateral projections). Morphometric analysis of the slow-motion video files was performed with MMC Multimeter software (MMCSoft, Russia). Videomicroscopy records of the patients` respiratory ciliated cells are provided in Supplemental material section (Additional files 5–25: Movies S1–S21).

### Targeted next generation sequencing

An Almazov custom gene panel probeset was used for screening of 206 genes implicated in isolated or syndromic congenital heart disease, heterotaxy, renal defects, motile and non-motile ciliopathies (Additional file 1). Probeset regions were extended by 100 bp in both directions to capture variants in non-coding areas during downstream analysis. The DNA-libraries were prepared with a SureSelect Target Enrichment kit (Agilent Technologies; Waldbronn, Germany), pooled together and analyzed using the MiSeq platform. Sarek v3.1 pipeline from nf-core was used for read processing and variant calling [35]. Reads were mapped to GRCh38 version of genome assembly. Briefly, for read processing GATK recommended workflow was implemented and following tools were used: nextflow v22.10.2, python v3.10.6, fastp v0.23.2, fastqc v0.11.9, bwa v0.7.17, bcftools v1.16, samtools v1.16.1, mosdepth v0.3.3, gatk4 v4.3.0.0, vcftools v0.1.16. For variant calling HaplotypeCaller v4.3.0.0 and Deepvariant v1.4.0 were used. Filtered variants were annotated with Variant Effect Predictor (VEP) v.104 using frequency data from gnomAD, TOPMed and ExAC databases. Additionally, CADD v1.6 and dbNSFP v4.2 databases were used to assign pathogenicity prediction scores to variants. To assess the influence of variants on splicing, we utilized Sequence Ontology (SO) classifications provided by VEP, along with precomputed SpliceAI scores from Gencode v24 by Illumina and dbscSNV v1.1 scores from dbscSNV. These resources were integrated as plugins within VEP. New ClinVar records were also incorporated during analysis. Before undergoing manual curation, variants were selected based on the following criteria: genotype quality > 20, coverage depth > 5 for SNPs and > 10 for InDels, AF in gnomAD < 0.01, CADD score > 20, non-Benign classification in ClinVar, protein sequence altering or splicing altering consequences. The identified genetic variants were classified according to American College of Medical Genetics and Genomics (ACMG) recommendation [36]. Verification of the NGS data, as well as target genetic analysis of the probands` relatives was done by Sanger sequencing using BigDye Terminator Sequencing Kit (Applied Biosystems) and Genetic Analyzer AB3100 (Applied Biosystems/Hitachi, Japan). Gene-specific primers were designed using NCBI Primer Blast tool (https://www.ncbi.nlm.nih.gov/tools/primer-blast/) (Additional file 2). The causative variants were submitted to the ClinVar database (https://www.ncbi.nlm.nih.gov/clinvar/), accession numbers SCV004176731–SCV004176752.

### Haplotype analysis

For haplotype analysis, SNP (single nucleotide polymorphism)-genotyping of the patients PCD-#03 II1, PCD-#04 II1, PCD-#-05 II1, and PCD-#06 II1 was carried out using Infinium Global Screening Array-24 v3.0 BeadChip, iScan System and Infinium HTS Assay Guide (Illumina, USA). To determine the prevalence of IBD in probands around the genomic region of interest, we used the program PLINK-1.9 (v1.90b6.21 64-bit [19 Oct 2020]) to estimate (p̂) for a window of 500 SNPs around the variant of interest (500 SNPs downstream and 500 SNPs upstream of the SNP). For a control comparison of the rate of p̂ decrease in the neighborhood of the variant of interest, we performed the same procedure for 200 randomly selected variants at nonoverlapping intervals on different chromosomes. The p̂ measurements were performed in 50 SNP increments on either side of the fixed variant, considering only highly confident HapMap variants.

### Immunofluorescence staining

Suspensions of the patients` respiratory epithelial cells were spread onto glass slides using an automated cytocentrifuge (Cyto-Tek; Sakura, Tokyo, Japan). The slides were air dried and stored at − 80 °C until use. The cells were fixed in 4% paraformaldehyde for 15 min and permeabilized with 0.5% Triton X-100 for 10 min at room temperature. Then, the cells were blocked with 15% FBS (Gibco) for 30 min followed by incubation with primary antibodies for 1.5 h and with secondary antibodies for 45 min also at room temperature. In particular, we used anti-DNAH5 rabbit polyclonal antibody (HPA037470, 1:300 in 1xPBS, Sigma-Aldrich, MO, USA) and anti-acetylated tubulin monoclonal antibody specific for ciliary axoneme (T7451, 1:1000 in 1xPBS, Sigma-Aldrich, MO, USA), goat anti-mouse Atto 550-conjugated secondary antibodies and goat anti-rabbit Alexa Fluor 488-conjugated secondary antibodies (1:1000 in 1xPBS, Thermo Fisher Scientific, USA). For cell nuclei visualization 4′,6-diamidino-2-phenylindole ((DAPI), Thermo Fisher Scientific, USA) was used at a concentration of 0.1 μg/mL. The slides were examined with Zeiss Axio Observer Z1 and ZEN Blue software (Carl Zeiss Microscopy, Jena, Germany).

### RNA extraction and reverse transcription-polymerase chain reaction (RT-PCR)

Total RNA was extracted from nasal-brush biopsy material of patients and healthy donors using the TRIzol Reagent (Invitrogen) according to manufacturer’s recommendations. First-strand cDNA was synthesized from 300 ng of total RNA with a MMLV RT kit (Evrogen, SK021, Russia). To test whether the c.2052+3G>T and c.3599-2A>G variants disrupt the splice sites in *DNAH5* gene*,* the c.10949-2A>G and c.1797C>G variants—in *HYDIN* gene, and the c.510+1G>C variant—in *ZMYND10* gene, cDNA fragments encompassing the corresponding transcript regions were amplified from patients` and control samples by standard PCR (annealing temperature 57 °C) with specific primers designed using NCBI Primer Blast tool (Additional file 2). For analysis of the *HYDIN* variants, the primers discriminating between the functional gene (chr 16q22.2) and its pseudogene *HYDIN2* (chr 1q21.1) sequences were designed. The PCR products were than analyzed by electrophoresis in a 1.5–3% agarose gel, as well as by Sanger sequencing (see above). For more careful evaluation of HYDIN transcripts alterations, the PCR products were subcloned using CloneJET PCR cloning kit (Thermo Scientific) with subsequent Sanger sequencing of the clones so that normal and mutated alleles could be analyzed separately.

## Results

The principal clinical features of the patients and the data on their cilia beat pattern are summarized in Additional file 3. The vast majority of the probands had recurrent oto-sino-pulmonary infections and suffered from chronic upper and/or lower respiratory tract diseases including otitis, protracted rhinitis and sinusitis, adenoiditis, purulent endobronchitis, bronchiectasis, and multiple episodes of pneumonia. About 40% of the affected individuals (10 out of 26 patients) had also laterality anomalies—situs viscerum inversus totalis, as well as cardiac phenotype ranging from minor structural or conduction anomalies to severe malformation including septal defects and aortic dysplasia. The data on fertility status was available only from some of older patients. Family members originated from and resided in different regions of Russia including Northwestern Federal District, Central Federal District, Udmurt Republic, Tatarstan, Far East, and Dagestan. The gene-panel sequencing of the probands proved to be effective and allowed to uncover disease-causing variants in the 19 out of 21 families investigated (Table 1). *DNAH5* was the most frequently mutated gene (6 reported families), which is in accordance with previously published data on the prevalence of *DNAH5* mutations in PCD-cohorts, estimated at 15–30% [13, 37]. Novel or rare variations in *DNAH11,* another dynein axonemal heavy chain gene, were detected in 3 families. Damaging variants in *CFAP300* and *LRRC6* were each revealed in 2 families, while 7 genes including *ODAD4*, *ZMYND10, HYDIN*, *DNAL1, CCDC103,* and *OFD1* were affected once.

**Table 1:** Causative genetic variants in ciliary genes uncovered in the PCD-patients by high-throughput gene panel sequencing

Case ID/patient	Gene	Exon/intron	Genetic variant	rsID	gnomAD	Zygosity	Inheritance
Case 1 PCD-#01 II1	*DNAH5*	Ex50	chr5:13791995–13792002, NM_001369.3:c.8440_8447del; p.Glu2814*	rs755136231	ƒ = 0.0000131	Comp. het	pat
		Ex63	chr5:13753290, NM_001369.3:c.10815del; p.Pro3606Hisfs*23	rs397515540	ƒ = 0.000191		mat
Case 2 PCD-#02 II1	*DNAH5*	Ex63	chr5:13753290, NM_001369.3:c.10815del; p.Pro3606Hisfs*23	rs397515540	ƒ = 0.000191	Comp. het	mat
		Ex68	chr5:13735239, NM_001369.3:c.11653C>T; p.Arg3885*	rs756032160	ƒ = 0.0000197		pat
Case 3 PCD-#03 II1	*DNAH5*	Intr14	chr5:13901249, NM_001369.3: c.2052+3G>T	N/A	N/A	Comp. het	mat
		Ex51	chr5:13788752, NM_001369.3:c.8611 T>C; p.Phe2871Leu	rs138494768	ƒ = 0.0000657		N/A
Case 4 PCD-#04 II1	*DNAH5*	Intr14	chr5:13901249, NM_001369.3: c.2052+3G>T	N/A	N/A	Comp. het	pat
		Intr23	chr5:13871004, NM_001369.3:c.3599-2A>G	N/A	N/A		mat
Case 5 PCD-#05 II1	*DNAH5*	Intr14	chr5:13901249, NM_001369.3: c.2052+3G>T	N/A	N/A	Comp. het	pat
		Ex41	chr5:13820424, NM_001369.3:c.6763C>T; p.Arg2255*	rs745918507	ƒ = 0.0000263		mat
Case 6 PCD-#06 II1, II2	*DNAH5*	Intr14	chr5:13901249, NM_001369.3: c.2052+3G>T	N/A	N/A	Hom	mat/pat
Case 7 PCD-#07 II1	*DNAH11*	Ex21	chr7:21615171, NM_001277115.2:c.3910del; p.Arg1304Valfs*13	rs2128452309	N/A	Comp. het	mat
		Ex48	chr7:21739592_21739596, NM_001277115.2:c.7833_7837dup; p.Lys2613Serfs*14	N/A	N/A		pat
Case 8 PCD-#08 II2, II4	*DNAH11*	Ex23	chr7:21617754–21617758, NM_001277115.2:c.4231_4235del; p.His1411Alafs*13	N/A	N/A	Hom	mat/?
Case 9 PCD-#09 II1	*DNAH11*	Ex15	chr7:21600085, NM_001277115.2:c.2966G>A; p.Arg989Gln	rs1178187217	ƒ = 0.000013	Comp. het	mat
		Ex15	chr7:21599943, NM_001277115.2:c.2824C>A; p.Pro942Thr	rs189569144	ƒ = 0.000237		pat
Case 10 PCD-#10 II1	*C11ORF70 (CFAP300)*	Ex3	chr11:102058885–102058887, NM_032930.3:c.198_200delinsCC; p.Phe67Profs*10	rs1555069023	N/A	Hom	mat/pat
Case 11 PCD-#11 II1	*C11ORF70 (CFAP300)*	Ex3	chr11:102058885–102058887, NM_032930.3:c.198_200delinsCC; p.Phe67Profs*10	rs1555069023	N/A	Hom	mat/pat
Case 12 PCD-#12 II1, II2	*LRRC6 (DNAAF11)*	Ex2	chr8:132661558–132661559, NM_012472.6:c.79_80del; p.Ser27Valfs*13	rs769220870	ƒ = 0.000112	Hom	mat/pat
Case 13 PCD-#13 II1	*LRRC6 (DNAAF11)*	Ex5	chr8:132632957, NM_012472.6:c.436G>C; p.Asp146His	rs200321595	ƒ = 0.0000987	Hom	mat/pat
Case 14 PCD-#14 II1	*ZMYND10*	Ex1	chr3:50345533, NM_015896.4:c.47T>G; p.Val16Gly	rs138815960	ƒ = 0.000197	Hom	mat/N/A
Case 15 PCD-#15 II1	*CCDC103*	Ex4	chr17:44902549, NM_213607.3:c.461A>C; p.His154Pro	rs145457535	ƒ = 0.00125	Hom	mat/pat
Case 16 PCD-#16 II1	*HYDIN*	Intr64	chr16:70872181, NM_001270974:c.10949-2A>G	rs2143654921	N/A	Comp. het	mat
		Ex14	chr16:71069444, NM_001270974.2:c.1797C>G; p.Tyr599*	rs760517494	ƒ = 0.000269		pat
Case 17 PCD-#17 II1	*ODAD4*	Ex6	chr17:41938635, NM_031421.5:c.704dup; p.His235Glnfs*48	N/A	N/A	Hom	mat/pat
	*ZMYND10*	Intr5	chr3:50343306, NM_015896.4:c.510+1G>C	N/A	N/A	Het	pat
Case 18 PCD-#18 II1, II2	*DNAL1*	Ex2	chr14: 3654866–73654867, NM_031427.4:c.23_24del; p.Lys8Argfs*16	rs746219926	ƒ = 0.00002	Hom	mat/pat
Case 19 PCD-#19 IV1, III1	*OFD1*	Ex20	chrX:13767252, NM_003611.3: c.2725C>T; p.Arg909*	rs1060500123	N/A	Hem	mat

N/A, Not available; gnomAD, Allele frequencies in gnomAD Genomes database (Version:3.1.2); Comp.het, Compound heterozygote; Hom, Homozygote; Hem, hemizygote; mat, Maternally inherited; pat, Paternally inherited

### PCD cases associated with variants in dynein axonemal heavy chain genes

#### Common truncating loss-of-function variants in *DNAH5*

**Fig. 1:**
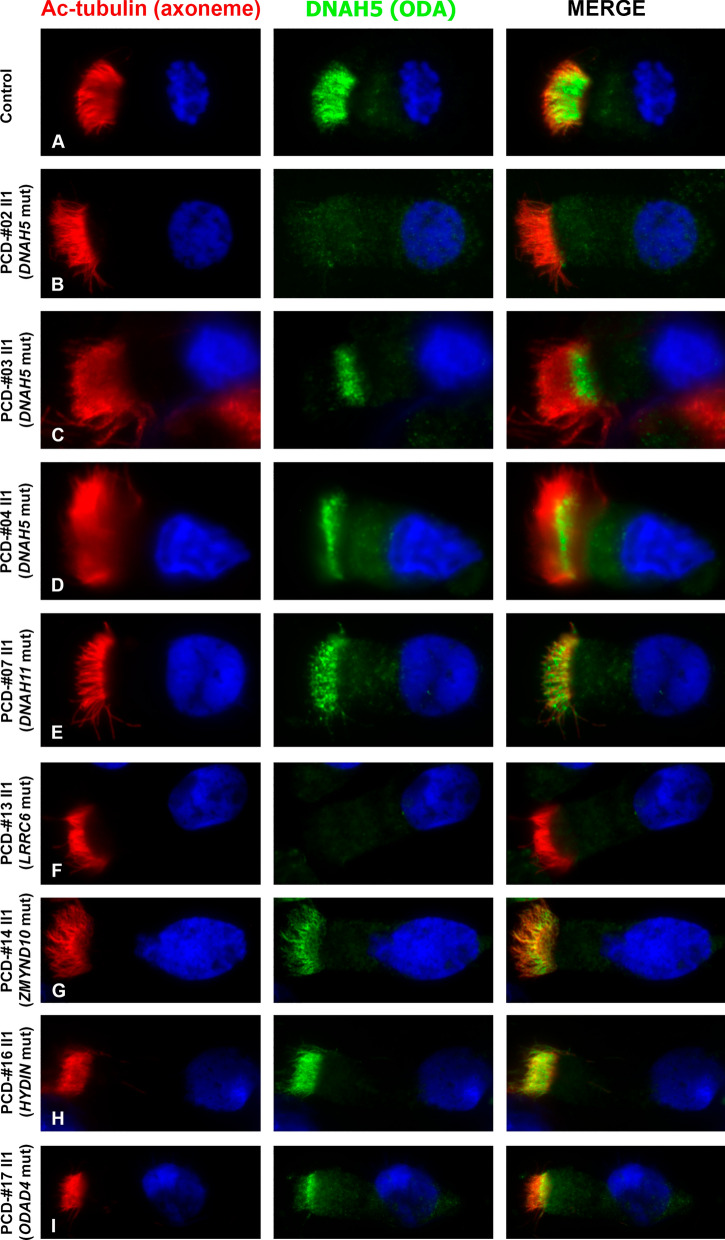
Localization of DNAH5 in respiratory epithelial cells from PCD-patients harboring pathogenic variants in different ciliary genes. Immunofluorescence staining of ciliated cells with antibodies against acetylated α-tubulin (marker of axoneme, red) and anti-DNAH5 antibodies (green). In contrast to a healthy control sample (**A**), complete absence of DNAH5 from ciliary axoneme was noted in patient PCD-#02 II1 with mutated *DNAH5* (**B**) and patient PCD-#13 II1 with mutated *LRRC6* (**F**). Mis-localization of DNAH5 was detected in patients PCD-#03 II1 and PCD-#04 II1 with mutated *DNAH5* (**C**, **D**), patient PCD-#14 II1 with mutated *ZMYND10* **(G)** and patient PCD-#17 II1 with mutated *ODAD4* (**I**). Seemingly normal pattern of DNAH5 distribution was noted in patient PCD-#07 II1 with mutated *DNAH11* (**E**) and patient PCD-#16 II1 with mutated *HYDIN* (**H**)

In two families, probands were compound heterozygotes for well-described truncating loss-of-function (LoF) variants in *DNAH5* (Additional file 4A, B). In the first case, a 7-year-old boy PCD-#01 II1 demonstrated upper airway disorders, bilateral bronchiectasis, and subtotal ciliary paresis in bronchial cells (Additional file 5). The boy combined a nonsense variant c.8440_8447del (p.Glu2814*) and a frameshift deletion c.10815del (p.Pro3606Hisfs*23) inherited from the father and mother, respectively. The second proband PCD-#02 II1, a 24-year-old man with typical Kartagener syndrome also showed immotile cilia or cilia with chaotic rigid strokes in nasal cells (Additional file 6). The patient possessed the same frameshift variant c.10815del, got from the mother, in combination with a heterozygous stop-gain variant c.11653C>T (p.Arg3885*), got from the father. IF analysis of the respiratory epithelium from PCD-#02 II1 confirmed the absence of DNAH5 protein in cilia, unlike the control sample (Fig. 1A, B). All the three truncating variants are predicted to induce a premature stop codon in the DNAH5 transcripts causing nonsense-mediated mRNA decay (NMD), and have low allele frequencies in the gnomAD (Table 1). The c.8440_8447del and c.10815del were also registered in the RUSeq, the database of genetic variants of Russian residents (http://ruseq.ru). The variations were previously classified as likely-pathogenic or pathogenic variants by ClinVar, and described as a genetic cause of PCD in patients of different ethnical groups in the literature (see “Discussion” section).

#### A novel splicing variant c.2052+3G>T in *DNAH5* uncovered in families from Udmurtia

**Fig. 2:**
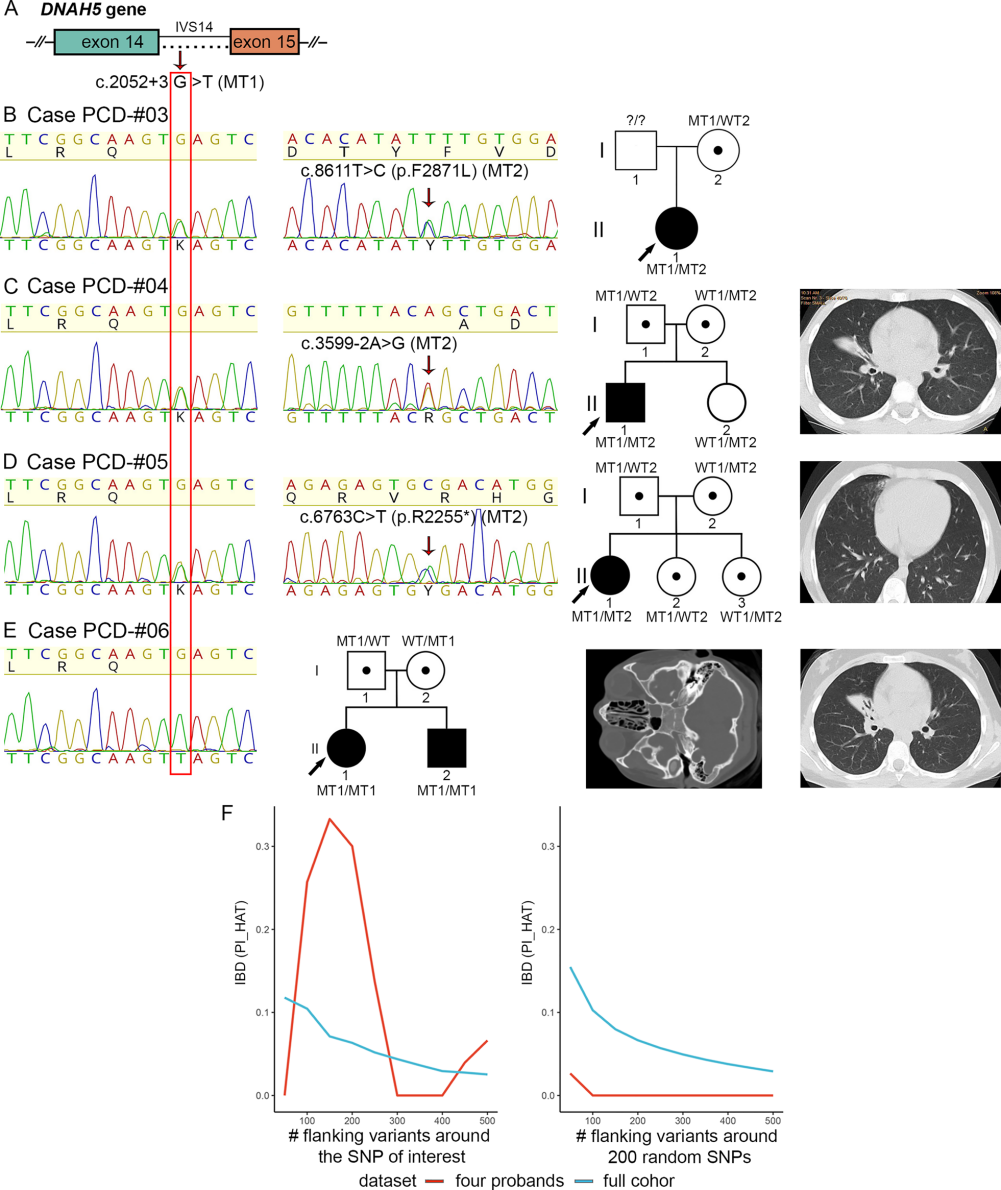
Identification of a novel *DNAH5* variant c. 2052+3G>T in four unrelated PCD families from Udmurtia. **A** Schematic image of a *DNAH5* fragment comprising exon 14 with the c.2052+3G>T variant pointed by an arrow and marked by a red frame. **B** Case PCD-#03. On the left, Sanger sequencing of the proband PCD-#03 II1 demonstrates compound heterozygosity for the c.2052+3G>T (MT1) and c.8611T>C (p.F2871L) (MT2). On the right, there is a family pedigree. **C** Case PCD-#04. On the left, Sanger sequencing of the proband PCD-#04 II1 demonstrates compound heterozygosity for the c.2052+3G>T (MT1) and c.3599-2A>G (MT2). In the middle, there is a family pedigree. On the right, computed-tomography (CT) scans of the proband with atelectasis and bronchiectatic lung disease. **D** Case PCD-#05. On the left, Sanger sequencing of the proband PCD-#05 II1demonstrates compound heterozygosity for the c.2052+3G>T (MT1) and c.6763C>T (p.R2255*) (MT2). In the middle, there is a family pedigree. On the right, CT scans of the proband with subpleural fibrous changes and bronchiectasis. **E** Case PCD-#06. On the left, Sanger sequencing of the proband PCD-#06 II1 demonstrates homozygosity for the c.2052+3G>T (MT1). In the middle, there is a family pedigree. On the right, CT scans of the proband`s lungs showing bronchiectatic disease and paranasal sinuses demonstrating sphenoiditis and ethmoiditis. **F** Change in identity by descent (IBD) as a function of increasing interval (step equals 50 SNPs downstream and upstream of the locus). In a range from 0 to 1, the p̂ (PI_HAT) metric indicates the proportion of alleles that are identical by descent. A value of 0 signifies that individuals are unrelated in terms of recent common ancestry, while a value of 1 means that individuals are genetically identical by IBD, such as identical twins or clones. The left panel shows the change in p̂ around NC_000005.10:g.13901249C>A (c.2052+3G>T) with increasing number of SNPs included in the analysis, for the four probands and the full cohort. Similarly, the right panel shows the mean change in p̂ around 200 randomly selected non-overlapping (within 500 SNPs) intervals, for the four probands and the full cohort. The analysis revealed a shared haplotype surrounding the *DNAH5* gene in four probands PCD-#03–06 II1 carrying the c. 2052+3G>T variant

Four families (PCD-#03, PCD-#04, PCD-#05, and PCD-#06) carried the intronic variant c.2052+3G>T in *DNAH5,* representing a single-nucleotide substitution at the exon 14/intron 14 junction (Table 1, Fig. 2A). Three out of the four probands were compound heterozygotes for the c.2052+3G>T and another damaging *DNAH5* variant, while one of them was a c.2052+3G>T homozygote*.* The c.2052+3G>T variant alters the third base of intron 14 (the donor splice site region), and, to our knowledge, it has not been previously described either in public databases of genetic variations (GnomAD, ClinVar, RUSeq), or in the literature. Among the affected probands, a 7-year-old girl PCD-#03 II1 with chronic adenoiditis, atelectasis and bronchiectasis showed asynchronic cilia beating with impaired mechanics of the stroke (Additional file 7). She had the c.2052+3G>T variant, inherited from the mother, in combination with a missense VUS c.8611T>C (p.Phe2871Leu), the origin of which could not be determined exactly (Fig. 2B). The c.8611T>C has a low population allele frequency in gnomAD and RUSeq, multiple computational predictions supporting its pathogenic effect on the gene product, and two ClinVar records describing the variant in PCD-individuals with conflicting interpretations of pathogenicity. Consistent with genetic findings, IF staining of the proband`s ciliated cells showed abnormal DNAH5 protein distribution within cilia with accumulation of the fluorescent signal in the peribasal area (Fig. 1C).

The second proband PCD-#04 II1 was an 8-year-old boy who presented with recurrent rhinosinusitis, bronchitis, bilateral tubootitis, and bronchiectasis. The most of cilia were immotile or showed stiff disordered movements (Additional file 8); the incorrect distribution of DNAH5 along the ciliary axoneme was also observed (Fig. 1D). The patient harbored a paternally inherited c.2052+3G>T variant *in trans* with a maternally inherited c.3599-2A>G variant located in intron 23 (Fig. 2C). The c.3599-2A>G is predicted to disrupt the canonical splice acceptor site, is not present in international public databases, and has high scores of deleteriousness. Notably, the variant has a low allele frequency (ƒ ~ 0.00021) in the national RUSeq database, being detected in two affected donors from European Russia.

In the third case, a 13-year-old girl PCD-#05 II1, who is the eldest of three daughters in the family, suffered from chronic rhinitis and adenoiditis, bilateral otitis media, as well as bronchiectasis and recurrent pneumonia. HSVM analysis demonstrated subtotal ciliary paresis with chaotic and rigid strokes of individual cilia (Additional file 9). The girl combined the c.2052+3G>T variant with a stop-gain substitution c.6763C>T (p.Arg2255*) (Fig. 2D), previously reported as pathogenic by ClinVar (ID 454795). Her clinically unaffected father and middle sister PCD-#05 II2 were heterozygous carriers of the c.2052+3G>T, while the mother and the youngest sister PCD-#05 II3 carried the c.6763C>T. Finally, the homozygous c.2052+3G>T was found in a non-consanguineous family PCD-#06 with two siblings (Fig. 2E). The proband PCD-#06 04 II1, a 13-year-old girl, had chronical sinusitis and lung disorders (Fig. 2E). HSVM showed altered mechanics of cilia including chaotic rigid strokes with incomplete extension of the axoneme (Additional file 10). Her younger brother PCD-#06 II2 was known to have the Perthes disease; the data on his oto-sino-respiratory manifestations were largely unavailable.

Remarkably, all the four families carrying the c.2052+3G>T variant were the residents of the same region of the country, namely, of Udmurt Republic, and were unrelated to each other. In all four cases, the c.2052+3G>T variant was not a de novo substitution, but was inherited by the probands from their parents. This seemed to suggest that the variant could rather represent an ancestral founder mutation in Udmurt population. By SNP-genotyping of the probands, we compared their haplotypes across the entire genome and, specifically, around the *DNAH5* locus (Fig. 2F). In particular, the region of interest included a maximum of 500 SNPs flanking the genomic variant NC_000005.10:g.13901249C>A (c. 2052+3G>T). The analysis confirmed that the patients had no genetic relatedness across the entire genome, but shared a haplotype of about 250–300 SNPs (~ 6.25 Mb) at the *DNAH5* locus, indicating a role of the founder effect in the spread of this intronic variant in Udmurtia.

**Fig. 3:**
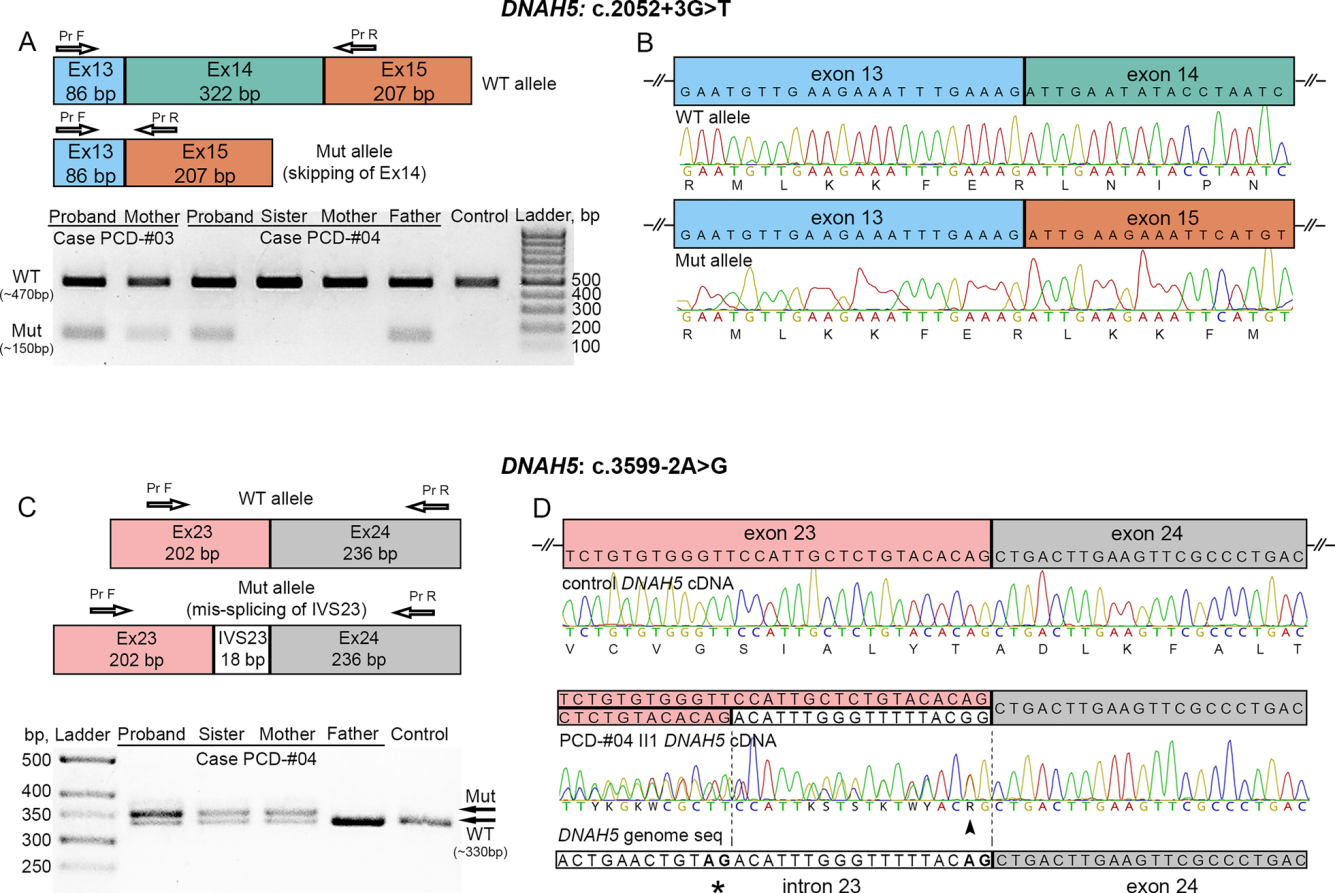
The intronic variants c.2052+3G>T and c.3599-2A>G result in abnormal splicing of DNAH5 transcripts. **A**–**D** Analysis of DNAH5 transcripts from nasal respiratory epithelial cells of the probands PCD-#03 II1 and PCD-#04 II1 and their relatives. **A** On the top: schematic results of PCR amplification of the DNAH5 cDNA region covering exons 13–15, that demonstrate a larger WT allele and a smaller mutant allele in carriers of the c.2052+3G>T variant. From below: gel-electrophoresis of RT-PCR samples from the PCD-#03 family (the proband and her mother), the PCD-#04 family (the proband and his unaffected sister, mother and father) and a control sample. **B** Sanger sequencing confirmed that the 472-bp fragment corresponded to the WT allele (on the top), while a smaller mutant fragment (150 bp) resulted from the skipping of exon 14 (from below). **C** On the top: schematic results of PCR amplification of the DNAH5 cDNA region comprising exons 23 and 24, that demonstrate a WT allele and a slightly larger mutant allele due to an 18-bp insertion in carriers of the c.3599-2A>G variant. From below: gel-electrophoresis of RT-PCR samples from the PCD-#04 family (the proband and his unaffected sister, mother and father) and a control sample. **D** Sequencing verified that the control fragment corresponded to the WT allele, while the proband`s sample represented a mix of the WT allele and altered transcript sequences harboring an 18-bp insertion between exon 23 and 24 (dashed lines), which corresponded to the 3’ part of the intron 23 sequence. From below, a *DNAH5* genomic region comprising an intron 23/exon 24 junction is shown. A canonical (AG) and a presumed cryptic (AG with an asterisk) splice acceptor sites are in bold

To specify the functional effect of the intronic variant c.2052+3G>T, we analyzed the DNAH5 transcripts from respiratory epithelium of the probands PCD-#03 II1 and PCD-#04 II1 and their relatives (Fig. 3). In particular, a 472-bp cDNA region covering exon 14 and flanking sequences was evaluated by RT-PCR with subsequent gel electrophoresis. In contrast to a control and samples of the PCD-#04 proband`s sister and mother, where only one band corresponding to a wild-type (WT) allele was detected, both patients’ samples showed two different fragments presumably representing a WT allele (~ 470 bp) and a smaller mutant allele (~ 150 bp), in accordance with heterozygosity (Fig. 3A). The two bands were also revealed in the PCD-#03 proband`s mother and PCD-#04 proband`s father, which is consistent with segregation of the c.2052+3G>T variant in the families. Sequencing of the PCR products confirmed that the 472-bp fragment corresponded to the WT allele, while a smaller fragment comprised the fused exon 13 and 15, lacking the exon 14 sequence (Fig. 3B). We conclude that the c.2052+3G>T affects canonical splicing and results in skipping of exon 14 (322 bp in size), which predicts a premature stop signal after the four amino acids changed due to out-of-frame deletion.

We also investigated the functional impact of the c.3599-2A>G variant on DNAH5 transcripts in the PCD-#04 family. In the control and paternal samples, a single band of ~ 330–340 bp was detected in an agarose gel, which apparently corresponded to wild-type alleles (Fig. 3C). The proband, his mother and sister showed two adjacent bands differing in size by 15–20 bp. Thus, the results did not reveal skipping of exon 24 (236 bp in size) but allowed to suspect some other transcript alteration. Target sequencing confirmed that the control sample corresponded to a WT allele (Fig. 3D). The patient`s PCR product indeed represented a mix containing WT and abnormal DNAH5 transcript sequences. The altered transcripts harbored the c.3599-2A>G substitution (arrowhead) and an 18-bp insertion between exon 23 and 24 which corresponded to the 3’ part of intron 23 and appeared to result from the utilization of a cryptic splice acceptor site (AG in bold with an asterisk) within intron 23 instead of the normal exon 24 splice acceptor (AG in bold). Such in-frame insertion is predicted to extend the protein by 6 amino acids but preserve a reading frame.

#### Novel and earlier reported genetic variants in *DNAH11*

The cohort included three cases of *DNAH11*-related PCD. An 11-year-old girl PCD-#07 II1, who suffered from chronic upper airway disorders, otitis media, and atelectasis, proved to be a compound heterozygote for two frameshifts in *DNAH11* (Additional file 4C). The c.3910del (p.Arg1304Valfs*13) and c.7833_7837dup (p.Lys2613Serfs*14) variants are predicted to produce a premature termination codon and have no allele frequency in gnomAD; the c.3910del has two records in ClinVar with pathogenic interpretation (ID 1805022). The c.3910del and c.7833_7837dup were inherited by PCD-#07 II1 from unaffected mother and father, respectively, and, according to ACMG guidelines, should be classified as pathogenic variations.

The second proband, a 9-year old girl PCD-#08 II2, was born to consanguineous parents originated from Tajikistan and living in the north-east of Russia. Two of four children in the family had symptoms of ciliary dyskinesia: the proband had typical features of Kartagener syndrome, and her 6-month-old sister (PCD-#08 II4) presented with neonatal pneumonia and year-round cough. Both girls harbored a frameshift variant c.4231_4235del in a homozygous state, that is predicted to create a premature stop codon (p.His1411Alafs*13) (Additional file 4D). To our knowledge, this 5-bp deletion has not been previously reported either in healthy populations, or in association with PCD phenotype, and, based on ACMG criteria should be also regarded as pathogenic.

The third proband—a 14-year-old boy PCD-#09 II1, had classic PCD phenotype, situs inversus with a surgically corrected congenital heart disease. The gene-panel sequencing detected two rare missense VUSs in *DNAH11* as a compound heterozygote (Table 1). In particular, the c.2966G>A (p.Arg989Gln) variant which leads to replacement of a basic and polar arginine residue with a charge-neutral and polar glutamine residue, was inherited from the mother. It has a low allele frequency in gnomAD, and has a deleterious effect on the gene product according to particular computational prediction tools. Besides, the p.Arg989Gln in combination with another pathogenic *DNAH11* variant had been earlier reported in a PCD-patient in ClinVar (ID 453287). In our study, the patient PCD-#09 II1 possessed the p.Arg989Gln *in trans* with the c.2824C>A variant at evolutionary conserved amino acid position (p.Pro942Thr). The variant was detected with a low allele frequency in healthy populations (gnomAD, RUSeq) and repeatedly described in association with PCD with conflicting interpretations of pathogenicity (ClinVar, ID 359618).

PCD patients with *DNAH11* mutations usually demonstrate normal ciliary ultrastructure by TEM and correct distribution of ODAs protein based on IF analysis [38–40], as we saw in a nasal brush-biopsy of the proband PCD-#07 II1 (Fig. 1E). Respiratory ciliated cells from DNAH11-mutated patients generally show the characteristic nonflexible and dyskinetic or hyperkinetic CBP [40]. Accordingly, in the patients PCD-#07 II1, PCD-#08 II2, II4, and PCD-#09 II1 we predominantly detected respiratory cells with paralysis or hyperkynetic chaotic stiff movements of cilia with incomplete cycle of the stroke (Additional files 11, 12, 13).

### PCD cases caused by common mutations in dynein arm preassembly, transport and anchoring factors CFAP300, LRRC6, ZMYND10, and CCDC103

**Fig. 4:**
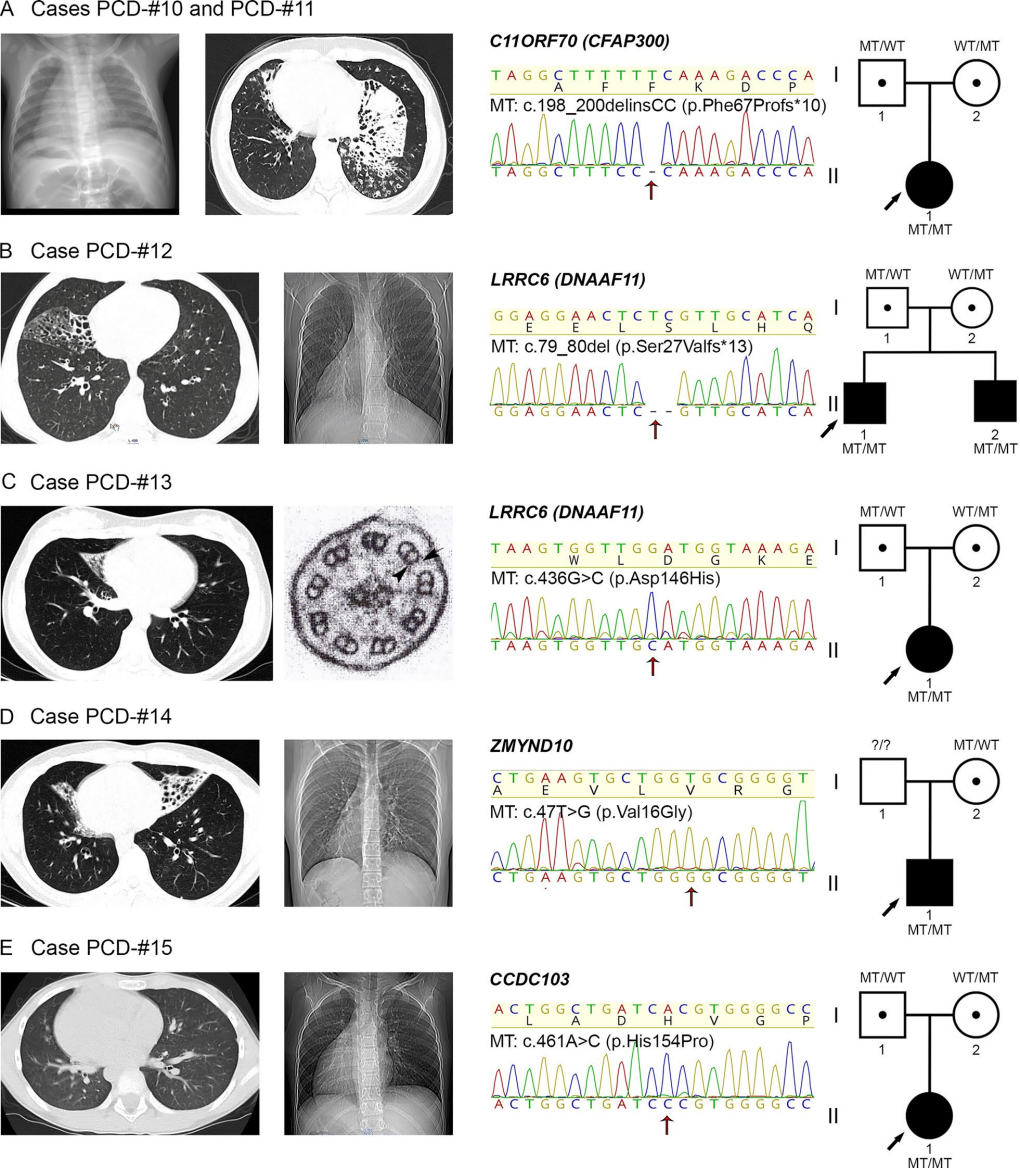
Clinical and genetic analysis of PCD families with common mutations in *CFAP300*, *LRRC6*, *ZMYND10* and *CCDC103*.** A** Cases PCD-#10 and PCD-#11. On the left, CT scans of the proband PCD-#10 II1 demonstrating situs inversus totalis and the proband PCD-#11 II1 showing a tree-in-bud pattern and fibrocystic transformation in the lungs. In the middle, Sanger sequencing of the PCD-#11 II1 demonstrates homozygosity for the c.198_200delinsCC variant in *CFAP300* (MT); the same variant is harbored by the PCD-#10 II1 (data not shown). **B** Case PCD-#12. On the left, CT scans of the proband PCD-#12 II1 illustrating bronchiectatic lung disease and his affected brother PCD-#12 II2 showing laterality defects. In the middle, Sanger sequencing of the proband confirms homozygosity for the c.79_80del variant in *LRRC6* (MT). **C** Case PCD-#13. On the left, there is a CT scan of the proband`s lungs showing bronchiectasis and an electron microscopy image of the axoneme cross-section which demonstrates the absence of ODA and IDA. In the middle, Sanger sequencing of the proband shows homozygosity for the c.436G>C (p.Asp146His) variant in *LRRC6* (MT). **D** Case PCD-#14. On the left, CT scans of the proband demonstrating bronchiectasis and dextrocardia. In the middle, Sanger sequencing of the proband verifies homozygosity for the c.47T>G (p.Val16Gly) variant in *ZMYND10* (MT). **E** Case PCD-#15. On the left, CT scans of the proband demonstrating situs anomalies. In the middle, Sanger sequencing of the proband confirms homozygosity for the c.461A>C (p.His154Pro) variant in *CCDC103* (MT). **A**–**E** On the right, family pedigrees are shown

The probands from two non-consanguineous families harbored a LoF homozygous variant c.198_200delinsCC, p.Phe67Profs*10 (Fig. 4A) in the highly conserved *CFAP300* gene encoding a protein essential for preassembly of ODA and IDA and transporting these complexes to motile cilia [24, 41, 42]. The c.198_200delinsCC is a frameshift variant combining deletion of TTT and insertion of CC, likely leading to NMD of the defective mRNA transcripts [24, 26]. The variant has several ClinVar records with pathogenic classification in association with PCD. The frameshift was found in a 2-year-old boy PCD-#10 II1 who did not have a prominent respiratory phenotype but demonstrated situs inversus. The second proband PCD-#11 II1 was a 38-year-old woman with typical Kartagener syndrome, who suffered from infertility and successfully gave birth to a son via in vitro fertilization. HSVM showed totally immotile cilia in the both probands (Additional files 14, 15) consistent with previous data [41, 42]. Targeted sequencing confirmed that the mutant *CFAP300* alleles were inherited by the probands from their heterozygous parents.

In other two families, we identified homozygous variants in *LRRC6* gene encoding a leucine-rich-repeat (LRR)-containing protein ensuring proper ODA and IDA assembly [43]. In a Caucasian family, a 14-year-old boy PCD-#12 II1, who demonstrated chronic maxillary sinusitis, adenoids, and bronchiectasis as well as total paralysis of ciliary apparatus (Additional file 16), harbored a 2-bp deletion variant c.79_80del in *LRRC6* (Fig. 4B)*.* The variant co-segregated with PCD-phenotype in the family: the proband’s younger brother PCD-#12 II2 presenting with Kartagener syndrome and totally immotile cilia was also homozygous for the c.79_80del with unaffected parents being heterozygous carriers. The c.79_80del is a frameshift variant predicted to create a premature termination codon (p.Ser27Valfs*13). It can be found in international and national population databases and has been classified as a pathogenic PCD-related variant in ClinVar and in the literature [44]. In another family, a 23-year-old woman PCD-#13 II1 suffering from regular upper airway infections, otitis media, and bronchiectasis had a missense variation c.436G>C (p.Asp146His) (Fig. 4C). The variant is also present in population databases with a low allele frequency (gnomAD, RUSeq) and has been repeatedly described in individuals with PCD in ClinVar and the publications (see “Discussion” section). Electron microscopy of ciliary axoneme cross-sections from the proband confirmed the absence of dynein arms (Fig. 4C)*.* Besides, the lack of ODAs was verified by IF staining with antibodies against DNAH5 (Fig. 1F). HSVM showed cilia to be completely immotile (Additional file 17). The results agree with the previous functional molecular studies which confirmed that PCD-patients with the Asp146His mutation demonstrated the reduced LRRC6 expression, absence of dynein arms, and abnormal cilia beating [45].

A 21-year-old male patient PCD-#14 II1, who had a history of regular upper airway disorders, adenoiditis, bronchiectasis, and s. inversus totalis with aortic valve dysplasia, possessed a missense variant c.47T>G (p.Val16Gly) in a homozygous state in *ZMYND10* gene (Fig. 4D). *ZMYND10* encodes another factor shown to be responsible for dynein arm preassembly and transport [46, 47]. The c.47T>G substitution has a low frequency in gnomAD and RUSeq, as well as multiple submissions to ClinVar in association with PCD phenotype, where it is classified as a pathogenic variation. IF staining showed the abnormal DNAH5 distribution within the cilia axonemes in proband’s respiratory cells (Fig. 1G). HSVM demonstrated a decreased number of cells with motor activity of cilia, asynchronic cilia beating with impaired mechanics (Additional file 18)*.* These results agree with the earlier obtained data concerning a phenotypic impact of different *ZMYND10* mutations, and, in particular, the homozygous c.47T>G substitution [46].

A girl at the age of 9 (PCD-#15 II1) presented recurrent otitis media, adenoiditis, chronic sino-pulmonary infections since birth, and situs inversus. The most of cilia from epithelial cells showed abnormal beating including asynchronism and low amplitude of strokes (Additional file 19). The girl had a homozygous missense variant c.461A>C in *CCDC103* gene; the mutant alleles were got from asymptomatic heterozygous parents (Fig. 4E). The gene encodes a coiled-coil domain containing-103 protein shown to be an essential factor for dynein-arm anchoring [48]. The c.461A>C leads to a change of a basic and polar histidine residue to a neutral and non-polar proline residue at codon 154 (p.His154Pro). According to gnomAD and RUSeq, the c.461A>C has a relatively high allele frequency among international and Russian populations. However, it was repeatedly described as a pathogenic variant in individuals with typical PCD phenotype including chronic respiratory tract infections and laterality defects [48, 49], as well as in patients with nonsyndromic asthenoteratozoospermia and total sperm immotility [50, 51]. Damaging effect of the genetic variant was proved by functional studies in zebrafish mRNA rescue experiments [48].

### Cases associated with variants in rare PCD-related genes *HYDIN*, *DNAL1*, *ODAD4*, and *OFD1*

#### A novel intronic variant c.10949-2A>G and nonsense variant c.1797C>G cause aberrant splicing of *HYDIN* transcripts

**Fig. 5:**
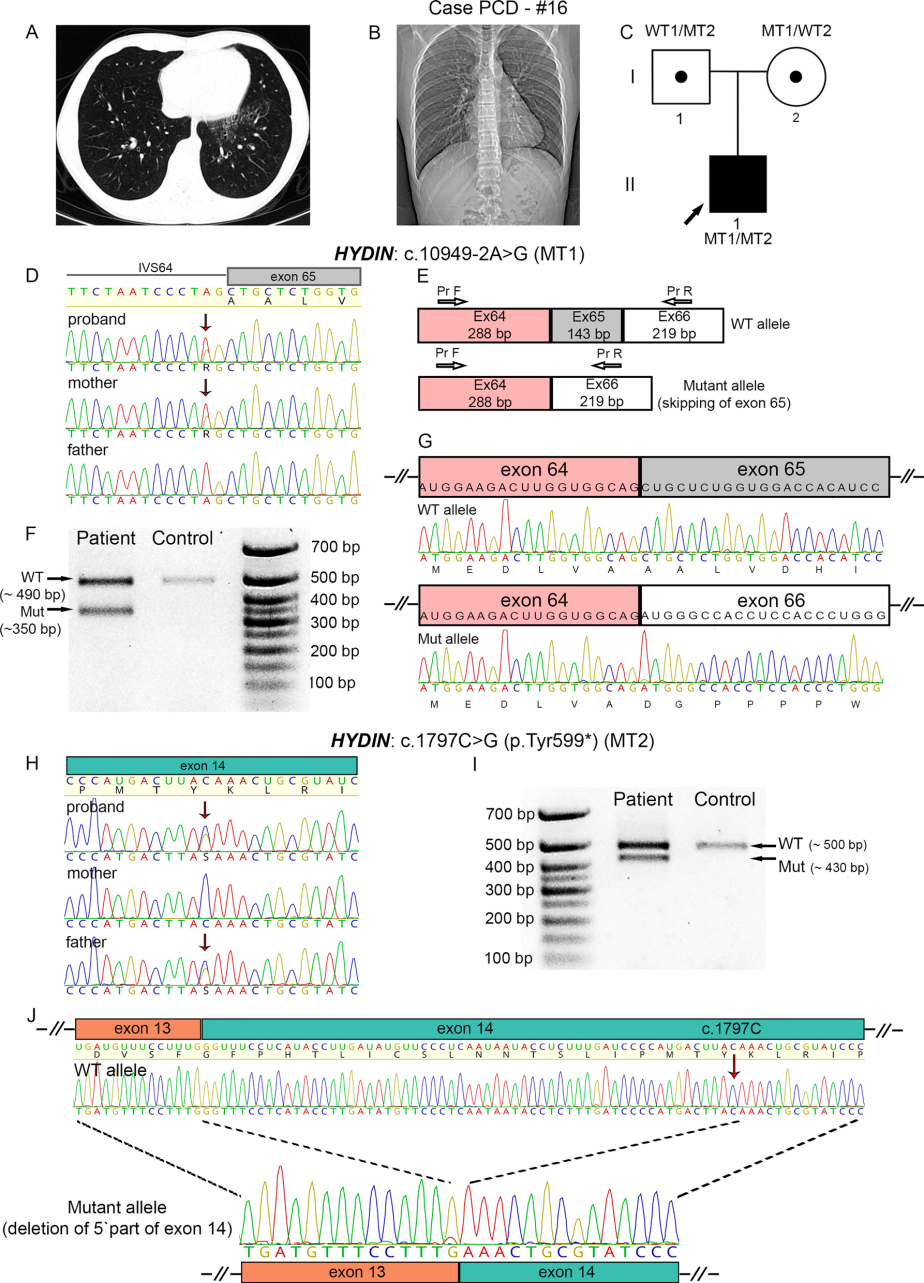
Identification of two loss-of-function variants in *HYDIN* and confirmation of their deleterious impact on splicing. **A**,** B** CT scans of the proband PCD-#16 II1 demonstrating situs solitus, atelectasis with bronchiectatic lung disease. **C** A family pedigree: the proband is a compound heterozygote for the c.10949-2A (MT1) and c.1797C>G (MT2) variants; the mother and the father are heterozygous carriers of MT1 and MT2, respectively. **D**–**G** Molecular characterization of the c.10949-2A variant. **D** Sanger sequencing results show that the proband and his mother possess the c.10949-2A as a heterozygote (pointed by arrows). **E** Schematic results of PCR amplification of the HYDIN cDNA region covering exons 64–66, that detect a larger WT allele and a smaller mutant allele in the proband carrying the c.10949-2A. **F** Gel-electrophoresis of RT-PCR samples from the patient and the control. **G** Sanger sequencing of RT-PCR products verified that a larger fragment (~ 490 bp) corresponded to the WT allele (on the top), while a smaller mutant fragment (~ 350 bp) resulted from the skipping of exon 65 (from below). **H**–**J** Molecular characterization of the c.1797C>G variant. Sanger sequencing results show that the proband and his father possess the c.1797C>G as a heterozygote (pointed by arrows). **I** Gel-electrophoresis of RT-PCR samples covering exons 13 and 14 of HYDIN cDNA from the patient and the control. **J** Sanger sequencing of RT-PCR products confirmed that a larger fragment (~ 500 bp) corresponded to the WT allele (on the top), while a smaller fragment (~ 430 bp) represented a mutant allele where the first 59 nucleotides of exon 14 sequence were skipped (from below)

A 15-year-old boy PCD-#16 II1, whose clinical manifestations included typical PCD symptoms and gastrointestinal anomalies (Additional file 3, Fig. 5A, B), harbored bi-allelic LoF variants in *HYDIN* encoding a central pair apparatus protein. The proband combined two heterozygous variants in *HYDIN*: an intronic variant c.10949-2A>G, inherited from the mother, and a nonsense variant c.1797C>G, inherited from the father (Fig. 5C, D, H). The c.10949-2A>G is predicted to affect a canonical acceptor splice site in intron 64. By now, it has not been registered in population databases, as well as in cohorts of PCD-patients. The second change c.1797C>G is located in exon 14 and predicted to create a TAC to TAG codon replacement (p.Tyr599*). A total gnomAD allele frequency for the variant is as high as 0.000269 with an increased frequency recorded in a Finnish population. Until this study, the variant has not been described in association with PCD cases. Consistent with the genetic findings, IF analysis showed normal distribution of DNAH5 protein along the cilia (Fig. 1H). HSVM demonstrated a considerably reduced number of cells with normal activity of ciliary apparatus, and non-coordinated stiff movements of cilia (Additional file 20).

To be sure that the c.10949-2A>G and c.1797C>G variants indeed affected *HYDIN* gene, and not the pseudogene *HYDIN2*, and to get more insights into their functional impact, *HYDIN* mRNA-transcripts from the respiratory epithelium of the individual PCD-#16 II1 were evaluated. In case of the c.10949-2A>G, a 490-bp cDNA region encompassing exon 65 and flanking sequences was analyzed (Fig. 5E). The patient’s PCR sample demonstrated two bands on a gel: a band of the same size as in the control (~ 490 bp) and a smaller band of ~ 350 bp, presumably lacking the exon 65 sequence (Fig. 5F). Sequencing verified that the larger fragment represented a WT allele, while the smaller mutant fragment comprised the fused exon 64 and 66 sequences (Fig. 5G). The results allowed to conclude that c.10949-2A>G disrupt the canonical acceptor splice site and leads to skipping of exon 65 (143 bp), which predicts a premature stop codon due to out-of-frame deletion. For functional analysis of the c.1797C>G, a cDNA fragment covering exon 14 and upstream sequences were evaluated. In contrast to a control PCR-product demonstrating a single fragment of expected size (~ 490–500 bp), the patient`s sample looked as double bands differing by ~ 50–60 bp (Fig. 5I). Sequencing confirmed that the larger band corresponded to a WT-allele, while the smaller fragment represented a mutant allele that lacked the first 59 nucleotides of exon 14 (Fig. 5J). Thus, a 3` border of this deletion corresponded to c.1797 mRNA position, where the C-to-G substitution occurred. These data allowed to hypothesize that the mutated locus could act as an ectopic splice acceptor site (AG, c.1796–1797 nucleotides), instead of the canonical splice acceptor site in intron 13. Such molecular events might cause aberrant splicing, resulting in an out-of-frame deletion of the 5` part of exon 14, which predicts a premature UAA stop signal after eleven novel amino acids.

#### The combination of a novel frameshift in *ODAD4* and a splicing variant in *ZMYND10*

**Fig. 6:**
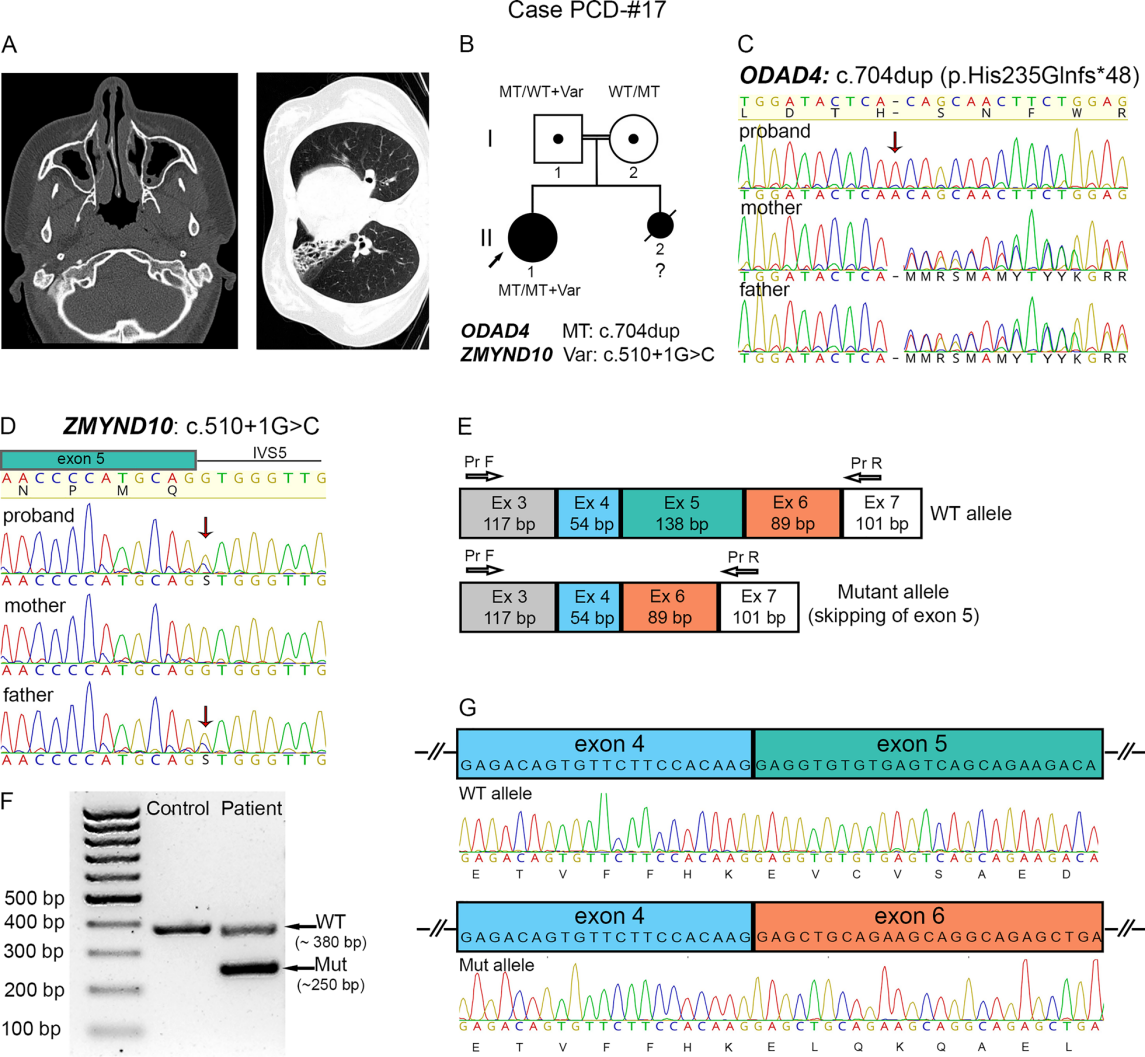
The PCD-#17 family combining a frameshift variant in *ODAD4* and a splice-site variant in *ZMYND10*.** A** CT scans of the proband PCD-#17 II1 demonstrating bilateral parietal thickening of the mucosa of the paranasal sinuses, polyposis and rhinosinusitis, as well as fibroatelectasis with bronchiectatic lung disease. **B** A family pedigree: the proband harbors a homozygous c.704dup variant in *ODAD4* (MT) and a heterozygous c.510+1G>C variant in *ZMYND10* (Var). **C** Sanger sequencing results demonstrate that the proband is homozygous for the c.704dup (p.His235Glnfs*48) variant in *ODAD4*, with the parents being heterozygous carriers of the variant. **D** Sanger sequencing results confirm that the proband and her father carry the c.510+1G>C variant in *ZMYND10*. **E**–**G** Analysis of ZMYND10 transcripts from nasal respiratory epithelial cells of the proband. **E** Schematic results of PCR amplification of the *ZMYND10* cDNA region covering exons 3–7 that demonstrate a larger wild-type (WT) allele and a smaller mutant allele. **F** Gel-electrophoresis of RT-PCR samples from the proband and the control. **G** Sanger sequencing verified that a larger fragment (~ 380 bp) corresponded to the WT allele (on the top), while a smaller fragment (~ 250 bp) resulted from the skipping of exon 5 (from below)

A 17-year-old girl PCD-#17 II1, presenting with polypous rhinosinusitis, fibroatelectasis and bronchiectasis, was born to consanguineous parents of Caucasian ethnicity, Dagestan region (Additional file 3, Fig. 6A). According to family history, the proband`s sister died on day 3 of life due to a critical CHD. NGS analysis of the patient revealed a novel frameshift variant c.704dup in *ODAD4* (*TTC25*) gene in a homozygous state, inherited from the heterozygous parents (Fig. 6B, C). The c.704dup is absent in public databases, represents a null variant that is predicted to create a premature translational stop signal (p.His235Glnfs*48). Although there are limited literature data on ODAD4 cellular function and clinical associations, the protein was recently described as a new member of the ODA-docking complex machinery in mice and humans [52]. Wallmeier et al. [52] showed that LoF variants in *ODAD4* resulted in reduced ciliary motility and underlie typical respiratory symptoms of PCD, and left–right body asymmetry randomization. Consistent with these findings, light microscopy of the proband`s respiratory epithelium demonstrated predominantly peribasal localization of DNAH5 in cilia and total cilia paralysis (Fig. 1I, Additional file 21).

Additionally, the patient PCD-#17 II1 proved to be a heterozygous carrier of a paternally inherited variant c.510+1G>C in *ZMYND10* gene (Fig. 6B, D). The variant has no allele frequency in gnomAD and RUSeq, and is predicted to disrupt a canonical donor splice site in intron 5. Sequence variations at the same genomic position, namely the c.510+1G>A and c.510+1Gdel variants had been previously submitted to ClinVar with a pathogenic/likely-pathogenic clinical interpretation (ID 645306, 2431468). We carried out analysis of ZMYND10 transcripts from ciliated epithelium of the patient, namely, the region of exon 5 and neighboring sequences (Fig. 6E). On a gel, a control PCR-product looked as one fragment of the expected size (~ 380 bp), seemingly corresponding to a WT allele (Fig. 6F). The patient`s sample showed two bands—a WT allele and a smaller fragment of ~ 250 bp, lacking exon 5 (138 bp), which was further confirmed by target sequencing (Fig. 6G). Thus, the substitution c.510+1G>C is a splicing mutation causing in-frame deletion of exon 5 and, therefore, shortening of the protein product by 46 amino acids.

#### A first described pathogenic frameshift variant c.23_24del in *DNAL1*

**Fig. 7:**
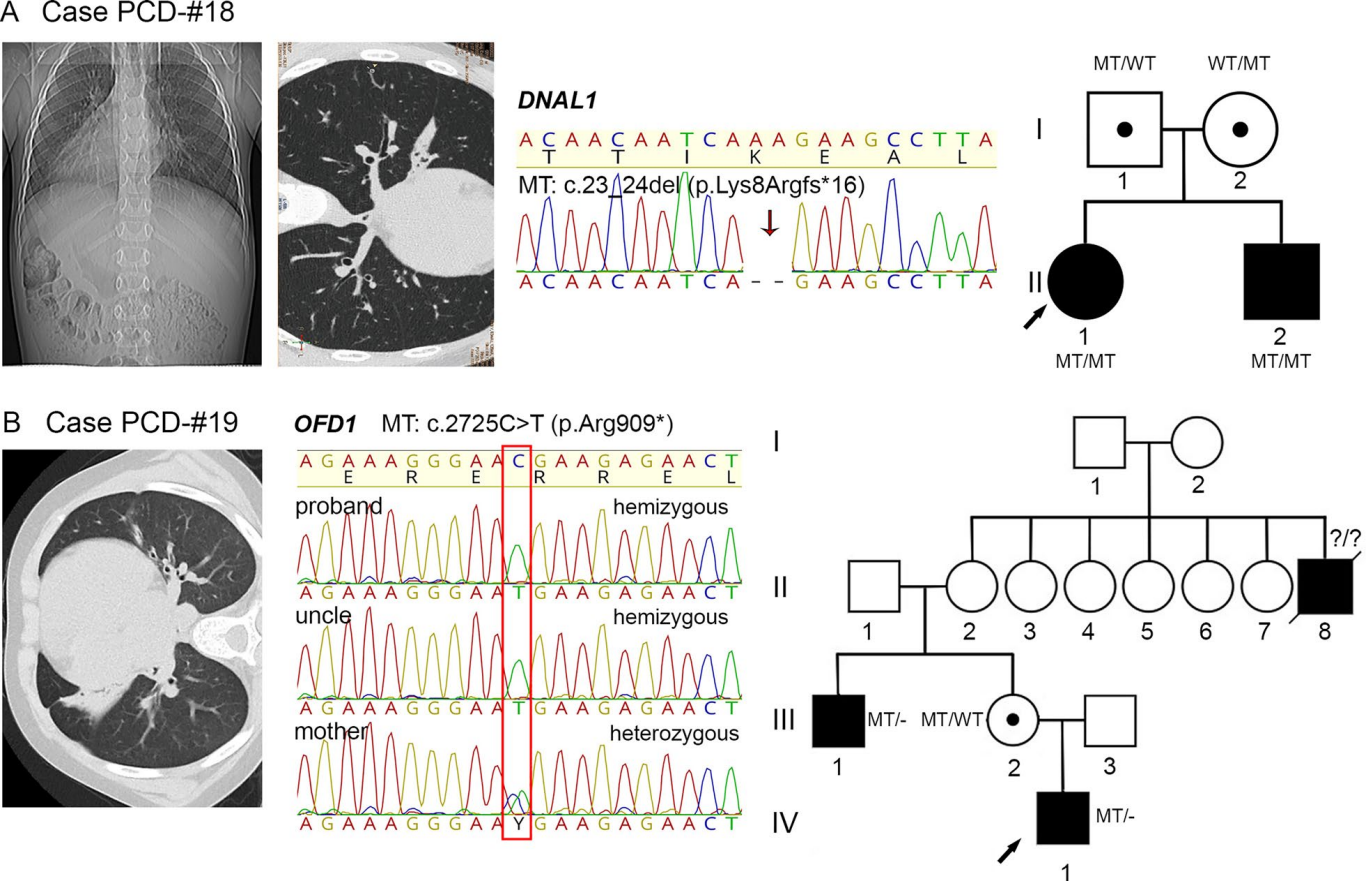
Identification of novel PCD-related loss-of-function variants in *DNAL1* and *OFD1* genes.** A** Case PCD-#18. On the left, CT scans of the proband PCD-#18 II1 with situs inversus totalis and a pattern of chronic bronchitis without bronchiectasis. In the middle, Sanger sequencing of the proband shows homozygosity for the c.23_24del (p.Lys8Argfs*16) in *DNAL1* (MT). On the right, a family pedigree: the proband and her affected brother are homozygotes; the parents are heterozygous carriers of the variant. **B** Case PCD-#19. On the left, CT scans of the proband PCD-#19 IV1 demonstrating bronchiectasis. In the middle, Sanger sequencing results show that the proband and his affected uncle PCD-#19 III1 are hemizygous for the c.2725C>T (p.Arg909*) variant in *OFD1* gene, with the proband`s mother PCD-#19 III2 being a heterozygous carrier of the variant (marked by a red frame); on the right, a family pedigree for four generations is shown

A proband, 5-year-old girl PCD-#18 II1, and her brother PCD-#18 II2 at the age of 7 were born to nonconsanguineous parents. Both children had respiratory symptoms characteristic for PCD; the proband also demonstrated situs inversus totalis (Additional file 3, Fig. 7A). Cilia from the siblings` biopsies were largely immotile; some of them showed chaotic asynchronic beating characterized by paralysis of the lower and middle thirds of the axoneme (Additional file 22). NGS and subsequent Sanger sequencing of the siblings revealed a homozygous pathogenic frameshift variant c.23_24del (p.Lys8Argfs*16) in *DNAL1* gene (Fig. 7A), the product of which is the ODA light chain 1 protein. It has a minor allele frequency in healthy populations, and, to the best of our knowledge, has not been previously described in PCD patients.

#### A rare familial *OFD1*-related primary ciliary dyskinesia

In one family, PCD symptoms were noted in the proband PCD-#19 IV1, a 12-year-old boy, and his uncle on the mother`s side (Additional file 3, Fig. 7B). The proband had a complex phenotype that included bradyarrhythmia, residual-organic CNS damage, congenital kidney defects (left-sided ureterohydronephrosis, megaureter, Fraley syndrome, and bilateral pyelectasis), hyperopia, and obesity of the first degree. No situs abnormalities or noticeable intellectual disability were detected. Severe oto-sino-pulmonary symptoms manifested since birth. HSVM of bronchial epithelium showed asynchronic ciliary beating with altered mechanics including a low amplitude of strokes and incomplete extension of the upper third of the axonemes (Additional file 23). The uncle PCD-#19 III1 suffered from chronic pneumonias, purulent obstructive bronchitis with a left sided lobectomy at the age of 7 in the anamnesis. He had no laterality defects; the data on his fertility status or the presence of any additional congenital malformations were unavailable. Besides, the death of a male child (PCD-#19 II8) at the age of 3 suffering from an unspecified lung disorder was registered among the family members, also on the mother`s side. Pedigree analysis made us suspect an X-linked form of PCD. Indeed, NGS of the proband detected a hemizygous nonsense variant c.2725C>T (p.Arg909*) in exon 20 of *OFD1* gene (Table 1, Fig. 7B), residing on chromosome Xp22.2 and encoding a univocal centrosome/basal body/centriolar satellites protein [53–56]. The same variant was confirmed in the affected uncle as a hemizygote and in the healthy mother—as a heterozygote. The c.2725C>T is absent in population databases and it has not been previously reported in the literature, but was submitted twice to ClinVar as a pathogenic variant in association with *OFD1*-related disorders (ID 404165).

### Genetically uncharacterized PCD

In two families with a suspected PCD diagnosis, the gene-panel sequencing proved to be uninformative: neither pathogenic/likely pathogenic variants nor VUSs have been detected. Both probands were the only children of their non-consanguineous parents, and no other cases of oto-sino-pulmonary disorders have been noted in their family history. One of the probands, a 13-year-old boy PCD-#20 II1 demonstrated normal situs but suffered from chronic polypous rhinosinusitis and had episodes of bronchitis and tubotitis. HSVM revealed mosaic changes in cilia motility (Additional file 24). Another proband, a 13-year-old boy PCD-#21 II1 had a Kartagener syndrome phenotype including chronic upper airway disorders, bronchiectasis, dextrocardia with an atrial septal aneurism. CBF and CBP evaluation also showed a reduced number of cilia and asynchrony of their beating in nasal biopsy cells (Additional file 25). Further in-depth genetic analysis such as exome or whole-genome sequencing might yield insights into the genetic basis of these PCD cases.

## Discussion

In the present study, we aimed to characterize a spectrum of causative genetic variants in 21 Russian families diagnosed with primary ciliary dyskinesia*.* In contrast to Europe and the United States, there is currently no a comprehensive Russian national registry of patients with PCD. While detailed clinical guidelines for diagnosis and treatment of Russian PCD patients has been developed, the recommendations on genetic testing are of a general nature and not adapted to Russian genetic background [57–59]. Due to significant genetic heterogeneity of PCD, a target genetic analysis of single genes, or, furthermore, selected ‘hot-spot’ exons and individual mutations, has not shown itself effective enough. Considering the high cost of high-throughput exome and whole-genome sequencing, utilization of well-designed multigene panels seems to be an optimal approach. Here we applied next-generation sequencing of a targeted panel comprising 40 PCD-related genes as well as additional genes associated with phenotypically-overlapping ciliopathies and heterotaxy syndromes. The gene-panel sequencing yielded good results with disease-causing variants being revealed in the 19 probands investigated (~ 90% of the cases). The genetic findings agreed well with particular observed anomalies of ciliary beat frequency and pattern detected by HSVM as well as intracellular distribution of ODA-specific DNAH5 protein.

### Identification of widespread mutations in ciliary genes

Based on NGS analysis, we revealed a number of genetic variants which were previously well-described as pathogenic variations associated with PCD in other ethnical groups and populations. As regards the structural genes, three families carried the *DNAH5* nonsense variants p.Glu2814Ter, p.Pro3606Hisfs*23, p.Arg3885Ter, and p.Arg2255Ter earlier reported as a frequent molecular-genetic cause of PCD in patients of different origin, including North American, German, Hungarian, Czech, Brazil and Italian individuals [13, 60–63]. Besides, the known deleterious variants were detected in *CFAP300*, *LRRC6, ZMYND10, and CCDC103* genes involved in proper ODA and IDA preassembly, transport or anchoring [41–43, 46–48]. That is, the probands from two unrelated non-consanguineous families possessed the homozygous frameshift variant c.198_200delinsCC affecting *CFAP300*. It has been previously found in PCD patients of different background including Israeli, German, Slavic and Finnish populations, and is believed to arise from an ancient European founder mutation [24, 26, 42]. Likewise, in yet another family we detected the homozygous missense variant c.47T>G (p.Val16Gly) in *ZMYND10*, which had also been suggested to represent a common European-founder-effect mutation [46]. The affected individuals from other two families harbored the homozygous causative variants in *LRRC6:* p.Ser27Valfs*13 and p.Asp146His, which had been repeatedly reported in Jewish, American and European cohorts [43–45]. Finally, one patient showed the homozygous missense substitution c.461A>C (p.His154Pro) in *CCDC103* which had been detected with a high prevalence in some populations, in particular, in the UK South Asian community [49]. The majority of the above-mentioned mutations have been also registered with some allele frequency in Russian healthy and affected donors within the RUSeq project, however, their exact prevalence in Russian population is to be determined.

### In-depth characterization of the DNAH5 splice site variant c.2052+3G>T typical for Udmurtia region

In the present research, we for the first time reported on the intronic variant c.2052+3G>T in *DNAH5,* which, according to sequencing analysis of mRNA transcripts from respiratory ciliated cells, caused abnormal splicing with an out-of-frame skipping of exon 14 (Table 1, Figs. 2, 3). Currently, the variant is absent from both international and Russian public databases on variant allele frequencies. Here, the c.2052+3G>T was surprisingly found in four families PCD-#03–06 not known to be related to each other. It is important to emphasize that all of them originated from the same region of Russia, namely from Udmurtia. Specifically, the probands and their parents were born and resided in Izhevsk City or the surrounding area. The Udmurt ethnos belongs to the eastern branch of Finno-Ugrian nations combining predominantly Caucasoid traits and some Mongoloid characteristics [64]. Previously, a comprehensive study on genetic structure of the Udmurt population has been carried out based on the data on genes of hereditary disorders, conditionally neutral DNA-polymorphisms, and abiotic parameters estimated by population statistic methods [65]. According to the demographic history of this region, the Udmurts have experienced twice a dramatic reduction in population size with a subsequent rapid growth within a short time period, which could favour genetic drift and a founder effect. This might explain a high prevalence rate of hereditary diseases noted in Udmurts as compared with other ethnic populations of Russia [65, 66]. Comparative haplotype analysis of the four affected probands PCD-#03-PCD-#06 II1 demonstrated that they shared a common haplotype across the *DNAH5* locus, which implies that the splice site variant c.2052+3G>T did not occur independently, but rather derived from a single ancestral event and might represent a founder mutation in Udmurt population. In practical terms, given the high prevalence of the c.2052+3G>T among Russian PCD patients from the Udmurt Republic, it should be considered when population-specific genetic tests are designed.

### Identification and functional assessment of other novel causative genetic variants

Apart from the c.2052+3G>T variant, NGS allowed to identify a number of other novel PCD-related genetic variations in *DNAH5*, *DNAH11*, *HYDIN*, *ODAD4*, *ZMYND10*, *DNAL1*, and *OFD1* genes. In particular, several more genetic variants identified in our cohort were proved to affect splicing. Namely, in the PCD-#04 family the c.3599-2A>G variant in *DNAH5* disrupted the canonical acceptor splice site and led to the in-frame 18-bp insertion of the 3’ part of intron 23 sequence in mRNA transcripts, apparently due to an activation a cryptic splice acceptor (Fig. 3). Besides, a case of the compound heterozygote for *HYDIN* deleterious variants c.10949-2A>G and c.1797C>G with “spliceogenic” effects was characterized in the PCD-#16 family (Fig. 5). The c.10949-2A>G variant affected the splice junction, which resulted in out-of-frame skipping of exon 65 with a predicted premature termination of translation. The c.1797C>G was initially classified as a nonsense substitution leading to a premature stop-codon (p.Tyr599*), however, the targeted cDNA analysis of the proband`s ciliated cells allowed to detect abnormally spliced transcripts lacking the 5′ part of exon 14 sequence upstream from the mutated site. These data suggest that the c.1797C>G variant could create an ectopic splice acceptor site (AG), probably stronger than the canonical one. Our data once again confirm the utility of in-depth transcripts investigations for better understanding of functional impact of nonsense variants and, as a consequence, molecular mechanisms of the disease pathogenesis. In practical terms, this knowledge can also have critical importance when applying read-through therapies of genetic diseases aimed at correcting the pathogenic effects of nonsense mutations [67, 68].

Of note, in one consanguineous family (PCD-#17), the proband combined previously unreported deleterious variants in two PCD-associated genes at once. In particular, along with a likely-pathogenic homozygous frameshift variant c.704dup (p.His235Glnfs*48) in *ODAD4*, the girl also carried the monoallelic intronic variant c.510+1G>C in *ZMYND10* gene. The latter was characterized as deleterious according to ACMG criteria, and experimentally proved to alter canonical splicing leading to in-frame exon 5 skipping (Fig. 7). Currently it is hard to determine unequivocally the contribution of this splice variant to the proband’s clinical phenotype, considering its heterozygous state and inheritance from the healthy father. Based on published data, di- or oligogenic inheritance is not common for PCD and other ciliopathies, though single cases have been reported. For example, digenic trans-heterozygous interactions between *DNAH6* and another PCD-related gene (*DNAH5* or *DNAI1*) were shown to cause airway ciliary dysfunction or heterotaxy [69]. Besides, a model of triallelic inheritance of BBS genes was suggested for Bardet-Biedl syndrome, digenic inheritance of unlinked ROM1 and RDS loci was described for retinitis pigmentosa, and digenic trans-heterozygous inheritance of *KIF14* and *TMEM67* genes—for Meckel Gruber syndrome [70–72]. To realize whether the proband PCD-#17 represents a rare case of triallelic inheritance (two mutant *ODAD4* alleles and one *ZMYND10* allele), the accumulation of data on similar cases and further functional studies on cell and animal models are needed.

Finally, we reported two families with LoF pathogenic variants in poorly characterized PCD-associated genes *DNAL1* and *OFD1*. In particular, two affected siblings in the PCD-#18 possessed a 2-bp frameshift deletion c.23_24del (p.Lys8Argfs*16) in *DNAL1*, encoding the ODA light chain 1 protein. *DNAL1* belongs to extremely rare PCD-related genes, with single pathogenic variants having been described in the literature. That is, the first detailly described variant was a missense change c.449A>G (p.Asn150Ser) [73]. Later, only several deletions of different size were reported [23], (ClinVar ID 662859, 228,252, 406,536, and 2,426,971). The male patient and his uncle from the family PCD-#19 harbored the hemizygous nonsense variant c.2725C>T (p.Arg909*) in *OFD1,* encoding a univocal centrosome/basal body/centriolar satellites protein vital for biogenesis of both primary and motile cilia [53–56]. Initially, OFD1 had been regarded as the morbid gene responsible for oral-facial-digital syndrome type I with X-linked dominant inheritance characterized by a specific clinical picture including dysmorphic facial features, malformations of the oral cavity and digits, and CNS damage (MIM #311200, [74]). Later, LoF variants in *OFD1* were determined as a genetic cause of several phenotypically variable and overlapping X-linked recessive conditions with multiorgan involvements, namely, Simpson-Golabi-Behmel syndrome type 2 (MIM # 300209), Jubert syndrome type 10 (MIM #300,04), isolated retinitis pigmentosa (MIM # 300424) [53, 75–79]. Since some affected individuals also presented with chronic respiratory disease with nasal cilia beating abnormalities, PCD was defined as part of an *OFD1*-related disorders spectrum [53, 79, 80]. Notably, our patient, who possesses the nonsense *OFD1* variant in exon 20, has a pronounced PCD phenotype with some additional pathologies which are however milder then severe neurological/renal/retinal/skeletal syndromic anomalies typical for OFD1-related syndromes. The data are in good agreement with previous observations indicating that truncating variants in a C-terminal part of OFD1 (especially in exons 16–22) nearly always resulted in respiratory phenotype [76, 81, 82]. Moreover, truncations in exons 20 and 21 were shown to cause respiratory phenotypes not associated with severe extra symptoms, which might be due to their damaging effect on ciliary motility but a limited impact on primary cilia function [81, 82].

## Conclusions

To conclude, the present study provides a comprehensive clinical and genetic characterization of 21 cases of primary ciliary dyskinesia, and represents one of the first detailed investigations on genetic spectrum of PCD in Russian population. The custom gene-panel sequencing proved to be highly efficient in detection of disease-related genetic variants in the patient cohort. The uncovered variations included common mutations, which had been previously reported as a genetic cause of PCD in other different populations, as well as novel variants, some of which probably specific for Russian patients. Additional functional analysis of mRNA transcripts from the patients` respiratory ciliated epithelium enabled us to verify and specify the detrimental impacts of the particular genetic variants in *DNAH5*, *HYDIN*, and Z*MYND10* on splicing process resulting in whole exon skipping, partial intronic insert or intraexonic deletion. In particular, we, for the first time, describe the splice site variant c.2052+3G>T, which leads to skipping of exon 14 in DNAH5 transcripts and is proposed to be an ancestral founder mutation in Udmurt population. We believe that the reported data make an important contribution to the study of genetic structure of primary ciliary dyskinesia in Russian population.

### Supplementary Information

Additional file 1: The SureSelect Custom Targeted Gene Panel. The panel comprises 206 genes implicated in isolated or syndromic congenital heart disease, heterotaxy, renal defects, motile and non-motile ciliopathies. The 40 genes described in association with Primary ciliary dyskinesiacases are highlighted in yellow. Additionally, 11 heterotaxy-related genes are highlighted in green

Additional file 2: Sanger sequencing primers for NGS data verification, target analysis of the probands’ relatives, and transcript analysis. The table includes forward and reverse PCR primers designed for Sanger sequencing. The primers were used for NGS data verification, targeted sequencing of DNA samples from the probands’ relatives, and analysis of cDNA samples obtained from nasal brush-biopsies

Additional file 3: Clinical characteristics of the PCD-patients and morphofunctional analysis of their respiratory cilia. The table provides information on clinical data of PCD-patients and the results of high-speed video microscopy of nasal/bronchial ciliated cells

Additional file 4: Clinical and genetic analysis of PCD families with common truncating mutations in *DNAH5* and novel loss-of-function variants in *DNAH11*. **A** Case PCD-#01. On the top panel: computed-tomographyscans of the proband's lungs showing a tree-in-bud patternand paranasal sinuses demonstrating chronic hyperplastic rhinosinusitis. On the lower panel: Sanger sequencing of the proband demonstrates compound heterozygosity for c.8440_8447deland c.10815delvariants in *DNAH5*. **B** Case PCD-#02. On the top panel: CT scans of the proband showing situs inversus and bilateral interstitial changes in lungs, atelectasis with bronchiectatic disease. On the lower panel: Sanger sequencing of the proband demonstrates compound heterozygosity for c.10815deland c.11653C>Tvariants in *DNAH5*. **C** Case PCD-#07. On the top panel: CT scans of the proband demonstrating maxilloethmoidal sinusitis, fibroatelectasis, and situs viscerum solitus. On the lower panel: Sanger sequencing of the proband confirms compound heterozygosity for c.3910deland c.7833_7837dupvariants in *DNAH11*. **D** Case PCD-#08. On the left, CT scans of the proband demonstrating bilateral bronchiectatic disease and total darkening of paranasal sinuses and ethmoid cells. On the top right, Sanger sequencing of the proband demonstrates homozygosity for c.4231_4235delvariant in *DNAH11*. **A**–**D** On the right, the family pedigrees are shown.

Additional file 5: HSVM of ciliated cells from the proband PCD-#01 II1

Additional file 6: HSVM of ciliated cells from the proband PCD-#02 II1

Additional file 7: HSVM of ciliated cells from the proband PCD-#03 II1

Additional file 8: HSVM of ciliated cells from the proband PCD-#04 II1

Additional file 9: HSVM of ciliated cells from the proband PCD-#05 II1

Additional file 10: HSVM of ciliated cells from the proband PCD-#06 II1

Additional file 11: HSVM of ciliated cells from the proband PCD-#07 II1

Additional file 12: HSVM of ciliated cells from the proband PCD-#08 II1

Additional file 13: HSVM of ciliated cells from the proband PCD-#09 II1

Additional file 14: HSVM of ciliated cells from the proband PCD-#010 II1

Additional file 15: HSVM of ciliated cells from the proband PCD-#011 II1

Additional file 16: HSVM of ciliated cells from the proband PCD-#012 II1

Additional file 17: HSVM of ciliated cells from the proband PCD-#013 II1

Additional file 18: HSVM of ciliated cells from the proband PCD-#014 II1

Additional file 19: HSVM of ciliated cells from the proband PCD-#015 II1

Additional file 20: HSVM of ciliated cells from the proband PCD-#016 II1

Additional file 21: HSVM of ciliated cells from the proband PCD-#017 II1

Additional file 22: HSVM of ciliated cells from the proband PCD-#018 II1

Additional file 23: HSVM of ciliated cells from the proband PCD-#019 II1

Additional file 24: HSVM of ciliated cells from the proband PCD-#020 II1

Additional file 25: HSVM of ciliated cells from the proband PCD-#021 II1

## Data Availability

Verified sequencing data for genetic variants reported in this manuscript are deposited into ClinVar (https://www.ncbi.nlm.nih.gov/clinvar/; accession numbers SCV004176731- SCV004176752). The data generated and analyzed during this study are shown in the article and supplementary information files. Additional data generated and/or analyzed during the current study are available from the corresponding author on reasonable request.

## Abbreviations

ACMG: American College of Medical Genetics and Genomics
CBF: Ciliary beat frequency
CBP: Ciliary beat pattern
DAPI: 4′,6-Diamidino-2-phenylindole
HSVM: High-speed video microscopy
IDA: Inner dynein arm
IF: Immunofluorescence
LoF: Loss-of-function
NMD: Nonsense-mediated mRNA decay
ODA: Outer dynein arm
PCD: Primary ciliary dyskinesia
SNP: Single nucleotide polymorphism
VUS: Variant of uncertain clinical significance
WT: Wild-type
