# Supplementary material for: Characterization of pathogenic genetic variants in Russian patients with primary ciliary dyskinesia using gene panel sequencing and transcript analysis
Source: Orphanet J Rare Dis. 2024 Aug 23;19:310. doi: 10.1186/s13023-024-03318-3 (PMC11344339; doi:10.1186/s13023-024-03318-3)
Supplement: Supplementary file 1 — Additional file 1: The SureSelect Custom Targeted Gene Panel. The panel comprises 206 genes implicated in isolated or syndromic congenital heart disease, heterotaxy, renal defects, motile and non-motile ciliopathies. The 40 genes described in association with Primary ciliary dyskinesiacases are highlighted in yellow. Additionally, 11 heterotaxy-related genes are highlighted in green [file 13023_2024_3318_MOESM1_ESM.pdf]

Additional file 1. The SureSelect Custom Targeted Gene Panel.

CPLANE1, ACTA2, ACTC1, ACVR2B, AHI1, ALMS1, ANKS6, ARL3, ARL6, ARMC, ARMC9, B3GAT3, B9D1, B9D2, BBS1, BBS10, BBS12, BBS2, BBS4, BBS5, BBS7, BBS9, BCOR, BICC1, BMP4, C11ORF70, C21ORF59, C5ORF42, CC2D2A, CCDC103, CFAP53, CCDC114, CCDC151, CCDC39, CCDC40, CCDC65, CCNO, CCP110, CDC5L, CDK13, CENPF, CEP104, CEP120, CEP164, CEP290, CEP41, CEP83, CFAP52, CFC1, CHD1L, CHD7, CRB2, CRELD1, CRKL, CSPP1, DHCR7, DNAAF1, DNAAF2, DNAAF3, DNAAF5, DNAH1, DNAH11, DNAH5, DNAH9, DNAI1, DNAI2, DNAJB11, DNAL1, DRC1, DSTYK, DYNC2H1, DYNC2LI1, DYX1C1, DZIP1L, EHMT1, EYA1, FGF20, FOXH1, FRAS1, FREM1, FREM2, GANAB, GAS8, GATA3, GATA4, GATA5, GATA6, GDF1, GJA1, GJA5, GLI3, GLIS2, GREB1L, HAND1, HNF1B, HYDIN, IFT140, IFT172, IFT27, IFT52, IFT74, IFT80, IFT81, INPP5E, INVS, IQCB1, ITGA8, JAG1, ANOS1, KIAA0556, KIAA0586, KIF7, LAMA2, LEFTY2, LRP2, LRRC56, LRRC6, LZTFL1, MCIDAS, MCTP2, MED13L, MEIS2, MKKS, MKS1, MMP21, MNS1, NEK1, NEK8, NKX2-5, NKX2-6, NME8, NODAL, NOTCH1, NOTCH2, NPHP1, NPHP3, NPHP4, NR2F2, NSD1, OFD1, PAX2, PBX1, PDE6D, PIH1D3, PKD1, PKD1L1, PKD2, PKHD1, PRKD1, PTK7, RBFOX2, REN, RET, ROBO2, RPGR, RPGRIP1L, RSPH1, RSPH3, RSPH4A, RSPH9, SALL1, SALL4, SDCCAG8, SIX1, SIX2, SIX5, SLIT3, SOX17, SPAG1, STK36, TAB2, TBC1D32, TBX1, TBX20, TBX3, TBX5, TCTN1, TCTN2, TCTN3, TFAP2B, TLL1, TMEM107, TMEM138, TMEM216, TMEM231, TMEM237, TMEM67, TNXB, TRAF3IP1, TRAP1, TRIM32, TTC21B, TTC25, TTC8, UMOD, UPK3A, WDPCP, WDR19, WDR34, WDR35, WDR60, WNT4, ZFPM2, ZIC3, ZMYND10, ZNF423

The panel comprises 206 genes implicated in isolated or syndromic congenital heart disease, heterotaxy, renal defects, motile and non-motile ciliopathies. The 40 genes described in association with Primary ciliary dyskinesia (PCD) cases are highlighted in yellow. Additionally, 11 heterotaxy-related genes are highlighted in green.
