# Supplementary material for: Characterization of pathogenic genetic variants in Russian patients with primary ciliary dyskinesia using gene panel sequencing and transcript analysis
Source: Orphanet J Rare Dis. 2024 Aug 23;19:310. doi: 10.1186/s13023-024-03318-3 (PMC11344339; doi:10.1186/s13023-024-03318-3)
Supplement: Supplementary file 2 — Additional file 2: Sanger sequencing primers for NGS data verification, target analysis of the probands’ relatives, and transcript analysis. The table includes forward and reverse PCR primers designed for Sanger sequencing. The primers were used for NGS data verification, targeted sequencing of DNA samples from the probands’ relatives, and analysis of cDNA samples obtained from nasal brush-biopsies [file 13023_2024_3318_MOESM2_ESM.docx]

**Additional file 2.** Sanger sequencing primers for NGS data verification, target analysis of the probands` relatives, and transcript analysis

| Gene (exon) | Forward primer | Reverse primer |
| --- | --- | --- |
| DNAH5 (14) | 5`-ACCCATCTGCCTTACAAAACAC-3` | 5`-CCCAAGTTAATAGTTTTGCCTACA-3` |
| DNAH5 (24) | 5`-ACTTCACTCCAGACCCAGTC-3`­ | 5`-TTTTGTGGGTTAGAGGGCAA-3` |
| DNAH5 (41) | 5`-GGTCACCACTACTTAAATGGGC-3` | 5`-GTGTTTCAATGTGGGGATTCTCTTA-3` |
| DNAH5 (50) | 5`-AAGCAGTAAAGAATACCCATGCT-3` | 5`-AAATTGGTGCCTCTGACACG-3` |
| DNAH5 (51) | 5`-AGAAACTCAACATCCATAAACTGAA-3` | 5`-CCTGAATTCCCAATTACGGCA-3` |
| DNAH5 (63) | 5`-ATGGCATTGGTAGAGATCAACAC-3` | 5`-CGGAAGGAAATGAAAGCCCG-3` |
| DNAH5 (68) | 5`-TGCAAAGGGCTTTCACAATAACTC-3` | 5`-CAGACAATTGAAAAGGGCAGTGG-3` |
| CFAP300 (3) | 5`-TCTGTACTTGGACACACAATGTTCA-3` | 5`-AAGCCAGCATAGTAGCAGTAGAC-3` |
| DNAAF11 (2) | 5`-TCTAAAAATAACACAGCACTTTCCA-3` | 5`-CTTATATTGAACAATGGGGACAGAA-3` |
| DNAAF11 (5) | 5`-GAGAATACAGTTTGCTGCTTTTAAC-3` | 5`-GACTTAAGCATTGACATTTTTGACA-3` |
| ZMYND10 (1) | 5`-ACTGAAGCCTAACCTGATCCTG-3` | 5`-AACTGTCCTGTCCCAGACTTTG-3` |
| ZMYND10 (5) | 5`-CTCAAATTCCATCAGCTCTGCCT-3` | 5`-TCCACAAGGTGAGGGACTATCT-3` |
| ODAD4 (6) | 5`-GTTTCAACAACTCCAAGCAGAGC-3` | 5`-TAGCTGACAGGGTAACTCCTCTTC-3` |
| HYDIN (14) | 5`-ACAGGTCTAGGACACACTCTATCA-3` | 5`-CTGCTGACACTGGACATGCT-3` |
| HYDIN (65) | 5`-GCTACTAGGTATGAGTGAGGGAT-3` | 5`-GGTTTCTCAAATATAGCCATCCAAG-3` |
| DNAH11 (15) | 5`-CAAAGCCAATCCCTCTCTGGA-3` | 5`-ACGGTCTTGCTATGCTGCTT-3` |
| DANH11 (21) | 5`-GCAGCTGAGTTTGCTCTTCTAA-3` | 5`-GCTCCACAAACTGCATATGTAAAAG-3` |
| DNAH11 (48) | 5`-TTACATTGACGCAGCTCCAG-3` | 5`-GGAATCCTAAGCTGTACTCGAA-3` |
| FOXJ1 (3) | 5`-AAGATGGCCTCCCAGTCAAAGT-3` | 5`-AGTGCTTCATCAAAGTGCCTCG-3` |
| DNAL1 (2) | 5`-GTTGTGGTGAGCGGAAATCA-3` | 5`-GTTCATGCATCCACCACCAT-3` |
| CCDC103 (4) | 5`-AAGATGAAGAGCTACAGGCTCC-3` | 5`-CATCAGTCAACCTGGTACAGC-3` |
| OFD1 (20) | 5`-AAGCAGGTCACAGAGTCAACTAA-3` | 5`-TTTGTACACCAATTGACTCCCCT-3` |
| NODAL (2) | 5`-TTAATAGCAAAGCTAGAGCCCTGT-3` | 5`-GCCATTGAGATTTTCCACCAGC-3` |
| cDNAH5 (14) | 5`-TTGCGGAAGTTCATGGATGTT-3` | 5`- CAAACAATTCCCCTGTGCCTG-3` |
| cDNAH5 (24) | 5`-ATTACACAGAGCCCCTTGCTT-3` | 5`-CAATGGAGATTTGCTCCTCCCT-3` |
| cHYDIN (65) | 5`-CCCAGAAGGTTGGGAGGATG-3` | 5`-TGGTCTGCAGGGAGCTGAAA-3` |
| cHYDIN (14) | 5`-TTTCAGTCCCAAGGAAGGCA-3` | 5`-GTGCCAGCTCGTATTTCTGC -3` |
| cZMYND10 (5) | 5`-GAAGGTGTTCCCTGTGTTCT-3` | 5`-CTAAGCATACGGCTCAAGGT-3` |
