# Supplementary material for: Characterization of pathogenic genetic variants in Russian patients with primary ciliary dyskinesia using gene panel sequencing and transcript analysis
Source: Orphanet J Rare Dis. 2024 Aug 23;19:310. doi: 10.1186/s13023-024-03318-3 (PMC11344339; doi:10.1186/s13023-024-03318-3)
Supplement: Supplementary file 4 — Additional file 4: Clinical and genetic analysis of PCD families with common truncating mutations in DNAH5 and novel loss-of-function variants in DNAH11. A Case PCD-#01. On the top panel: computed-tomographyscans of the proband's lungs showing a tree-in-bud patternand paranasal sinuses demonstrating chronic hyperplastic rhinosinusitis. On the lower panel: Sanger sequencing of the proband demonstrates compound heterozygosity for c.8440_8447deland c.10815delvariants in DNAH5. B Case PCD-#02. On the top panel: CT scans of the proband showing situs inversus and bilateral interstitial changes in lungs, atelectasis with bronchiectatic disease. On the lower panel: Sanger sequencing of the proband demonstrates compound heterozygosity for c.10815deland c.11653C>Tvariants in DNAH5. C Case PCD-#07. On the top panel: CT scans of the proband demonstrating maxilloethmoidal sinusitis, fibroatelectasis, and situs viscerum solitus. On the lower panel: Sanger sequencing of the proband confirms compound heterozygosity for c.3910deland c.7833_7837dupvariants in DNAH11. D Case PCD-#08. On the left, CT scans of the proband demonstrating bilateral bronchiectatic disease and total darkening of paranasal sinuses and ethmoid cells. On the top right, Sanger sequencing of the proband demonstrates homozygosity for c.4231_4235delvariant in DNAH11. A–D On the right, the family pedigrees are shown. [file 13023_2024_3318_MOESM4_ESM.pdf]

Additional file 4. Clinical and genetic analysis of PCD families with common truncating mutations in *DNAH5* and novel loss-of-function variants in *DNAH11*.

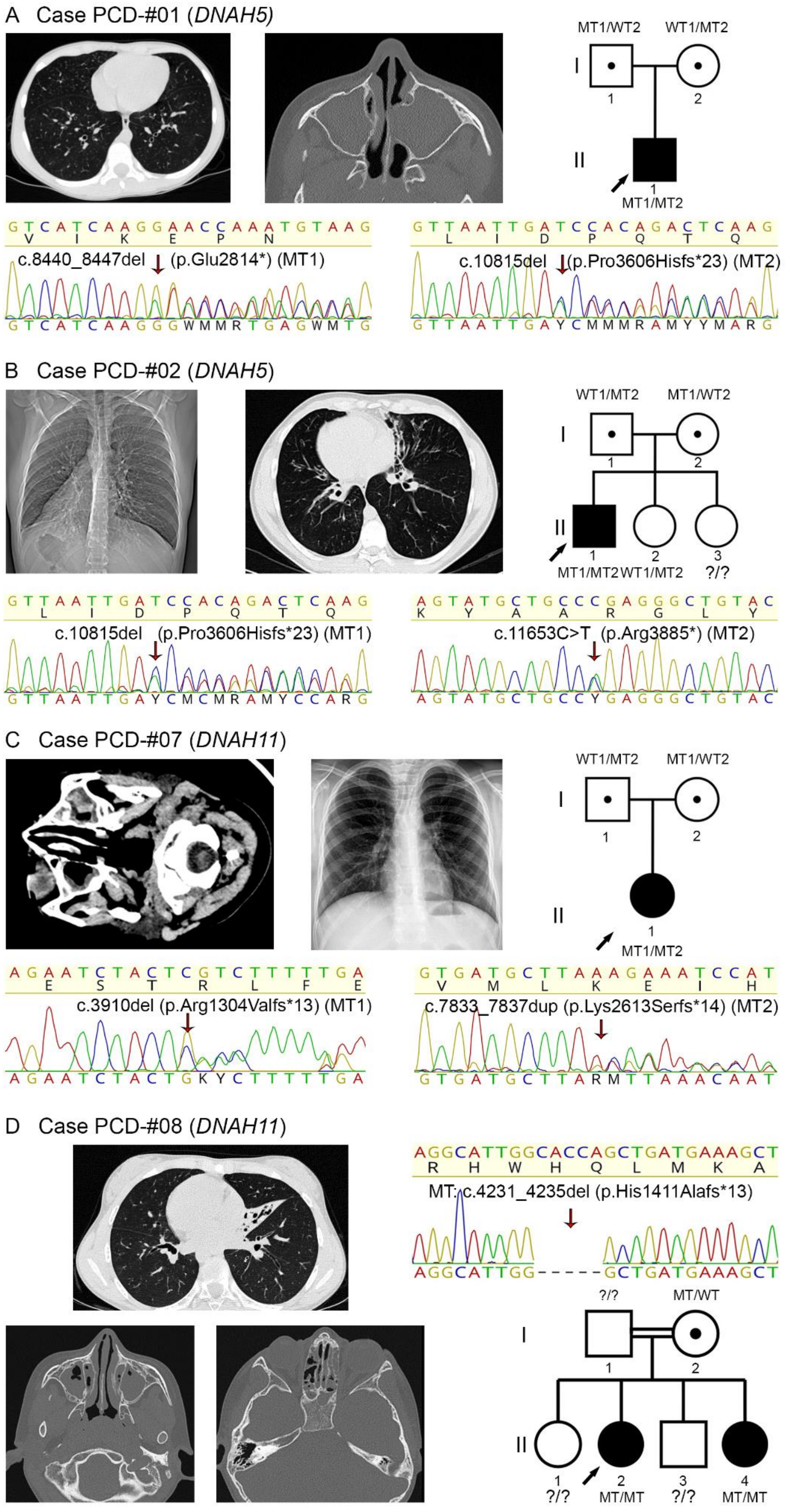

A Case PCD-#01. On the top panel: computed-tomography (CT) scans of the proband's lungs showing a tree-in-bud pattern (on the left) and paranasal sinuses demonstrating chronic hyperplastic rhinosinusitis (in the middle). On the lower panel: Sanger sequencing of the proband demonstrates compound heterozygosity for c.8440_8447del (MT1) and c.10815del (MT2) variants in *DNAH5*. **B** Case PCD-#02. On the top panel: CT scans of the proband showing situs inversus and bilateral interstitial changes in lungs, atelectasis with bronchiectatic disease (on the left, in the middle). On the lower panel: Sanger sequencing of the proband demonstrates compound heterozygosity for c.10815del (MT1) and c.11653C>T (MT2) variants in *DNAH5*. **C** Case PCD-#07. On the top panel: CT scans of the proband demonstrating maxilloethmoidal sinusitis, fibroatelectasis, and situs viscerum solitus (on the left, in the middle). On the lower panel: Sanger sequencing of the proband confirms compound heterozygosity for c.3910del (MT1) and c.7833_7837dup (MT2) variants in *DNAH11*. **D** Case PCD-#08. On the left, CT scans of the proband demonstrating bilateral bronchiectatic disease and total darkening of paranasal sinuses and ethmoid cells. On the top right, Sanger sequencing of the proband demonstrates homozygosity for c.4231_4235del (MT) variant in *DNAH11*. **A**-**D** On the right, the family pedigrees are shown.
